# Andrographolide Promotes Post-Ischemic Endothelial Angiogenic Activity via IL-10-Mediated M2-Like Microglia–Endothelial Crosstalk Involving Endothelial VEGFR2 Upregulation

**DOI:** 10.3390/biology15191715

**Published:** 2026-09-26

**Authors:** Hongfei Li, Hairong Xiang, Xinyu Li, Jiazhen Xie, Linfeng Zhao, Lan Yang, Riyi Shi, Yong Wang, Liangqin Shi

**Affiliations:** 1 College of Basic Medicine, Chengdu University of Traditional Chinese Medicine, Chengdu 611137, China; hongfeili123@hotmail.com (H.L.); 13036697356@163.com (H.X.); jiazhen18796@hotmail.com (J.X.); zlinfengzhao@hotmail.com (L.Z.); lanyang72@163.com (L.Y.); 2 School of Medical and Life Sciences, Chengdu University of Traditional Chinese Medicine, Chengdu 611137, China; 18050394729@163.com; 3 Center for Paralysis Research, Department of Basic Medical Sciences, College of Veterinary Medicine, Purdue University, West Lafayette, IN 47907, USA; riyi@purdue.edu

**Keywords:** M2-like microglia, angiogenic activity, ischemic stroke, Androprapholide, IL-10, VEGFR2

## Abstract

Ischemic stroke remains a leading cause of disability worldwide, and angiogenesis is critical for functional recovery. Although andrographolide (ANP) has anti-inflammatory and neuroprotective properties, its role in post-stroke angiogenesis remains unclear. Here, we investigated whether ANP promotes post-ischemic angiogenesis by modulating microglia–endothelial crosstalk. Using a ferric chloride (FeCl_3_)-induced carotid artery thrombosis model in male C57BL/6J mice, we found that ANP treatment (2.5 mg/kg) attenuated ischemic brain injury and neurological deficits, enhanced vascular stability, and improved cerebral blood perfusion. Mechanistically, ANP promoted a pro-reparative M2-like microglial state characterized by coordinated transcriptional changes, increased CD206 and IL-10, and reduced CD86 expression. ANP-treated microglia enhanced endothelial cell migration and tube formation while preserving tight-junction integrity through paracrine signaling. Co-immunoprecipitation and gain- and loss-of-function experiments further supported a microglial IL-10-dependent endothelial VEGFR2 upregulation. Together, these findings demonstrate that ANP promotes post-stroke reparative angiogenic activity through IL-10-mediated M2-like microglia–endothelial crosstalk involving endothelial VEGFR2 upregulation, linking its immunomodulatory effects to angiogenesis and highlighting microglia–endothelial crosstalk as a potential therapeutic target for ischemic stroke recovery.

## Simple Summary

Each year, millions of people worldwide are left with long-term disabilities after an ischemic stroke, and the promotion of vascular repair in the brain is critical for patient recovery. Andrographolide, an active anti-inflammatory component of the traditional Chinese herb *Andrographis paniculata*, has known beneficial properties, but whether it can improve post-stroke vascular remodeling remains unclear. Using an animal model of cerebral thrombosis, we found that andrographolide reduced brain injury, promoted vascular repair, and restored cerebral blood flow. In terms of how it works, andrographolide reprogrammed microglia into a repair-promoting state, enabling them to release signaling molecules that activated repair pathways in endothelial cells, enhancing migration, vessel formation, and barrier integrity. Our results show that andrographolide promotes post-stroke vascular repair, at least in part, by regulating the interaction between brain immune cells and vascular cells, offering new insight into how this compound may support brain repair after ischemic stroke.

## 1. Introduction

Stroke is the leading cause of long-term disability and the second most common cause of death worldwide. Approximately 30% of strokes are hemorrhagic (vessel rupture), while 70% are ischemic (arterial occlusion) [1]. Cerebral ischemia and hypoxia trigger a cascade of pathophysiological processes that ultimately result in neuronal damage. The clinical manifestations include motor deficits, speech impairment, confusion, and visual disturbances, all of which significantly impact patients’ quality of life [2].

To date, recombinant tissue-type plasminogen activator (rt-PA) is the only approved thrombolytic drug for acute ischemic stroke. However, rt-PA therapy must be administered within 4.5 h after stroke onset to achieve optimal therapeutic efficacy [3]. Additionally, thrombolysis increases the risk of intracerebral hemorrhage in patients. Even after successful thrombolysis, some patients still experience neurological dysfunction and permanent disability [4]. Therefore, it is important to develop novel therapeutic strategies for the subacute and chronic phases of ischemic stroke to improve clinical outcomes.

Among the various mechanisms governing post-stroke recovery, angiogenesis has emerged as a crucial therapeutic target. Indeed, enhanced microvascular density in the peri-infarct region correlates with prolonged survival and improved neurological outcomes in both stroke patients and experimental models [5,6]. Newly formed blood vessels not only restore blood flow to the ischemic area but also support neurogenesis, thereby facilitating functional recovery.

While promoting angiogenesis is widely regarded as a key strategy to enhance cerebral blood perfusion and neurological functional recovery after cerebral ischemia, its potential risks cannot be ignored. Studies suggest that pathological or dysregulated angiogenesis may be associated with impaired vascular maturation and compromised blood–brain barrier (BBB) integrity. For example, the overexpression of vascular endothelial growth factor (VEGF) in the acute phase, despite potently stimulating new vessel growth, can also lead to vascular leakage, cerebral edema, and even hemorrhagic transformation [7]. In recent years, there has been a growing consensus that promoting “functional angiogenesis,” defined as newly formed vessels with complete pericyte coverage and intact tight junctions, is crucial for successful stroke therapy [8,9]. Therefore, when developing pro-angiogenic therapeutic strategies, balancing the benefits against the risks and ensuring that the formation of mature, stable, and functional vascular networks remains a major challenge in the field.

Microglia, as innate immune cells of the central nervous system and a member of the macrophage system, undergo dynamic activation and functional reprogramming in response to ischemic stroke or other forms of injury [10]. Although these activation states have traditionally been described using the M1/M2 polarization framework [11], this binary classification does not fully capture the heterogeneous and dynamic nature of microglial states [12]. Accordingly, in the present study, we use the terms “M1-like” and “M2-like” as descriptive terms for activation-associated phenotypes rather than to imply discrete or fixed microglial states. M1-like microglia/macrophages are generally associated with pro-inflammatory responses and secondary brain injury, characterized by the production of inflammatory mediators such as ROS, TNF-α, IL-1β, MCP-1, and matrix metalloproteinases. These mediators can damage tight junction proteins and basement membrane components, aggravate blood–brain barrier breakdown, and promote leukocyte infiltration into ischemic brain tissue [13].

In contrast, M2-like microglia/macrophages are generally considered to exert anti-inflammatory, pro-reparative, and tissue-remodeling effects after ischemic stroke. They produce anti-inflammatory and repair-associated mediators, including TGF-β, IL-4, IL-10, and IL-13, which contribute to inflammation resolution, apoptotic cell clearance, extracellular matrix remodeling, and functional recovery [13,14,15]. In addition to their immunomodulatory effects, M2-like microglia/macrophages also participate in vascular repair [16,17] and release angiogenesis-related factors such as VEGF and IL-10 [18,19]. This raises the possibility that the activation of M2-like microglia facilitates angiogenesis after ischemic stroke. However, whether the pro-angiogenic microglia can be activated by exogenous drugs or signaling remains largely unknown.

Andrographolide (ANP; C_20_H_30_O_5_), the main active monomer component of Andrographis paniculata (*Burm.f.*) Nees, has been extensively studied and widely used in the prevention and treatment of various diseases, such as upper respiratory tract infections, bacterial dysentery, otitis media, and scabies, owing to its antibacterial, antiviral, immunomodulatory, and antithrombotic effects [20,21,22]. The existing research indicates that following ischemic stroke, andrographolide exerts potent neuroprotective effects through multiple mechanisms, including but not limited to the inhibition of neuroinflammation, neuronal apoptosis [23,24,25], oxidative stress, microglial and astrocytic activation [26,27], as well as the mitigation of blood–brain barrier disruption [28,29]. Beyond neurological contents, ANP has also shown to improve pathological vascular remodeling [30], repair the endothelial barrier [31] and regulate gastric vascular homeostasis [32]. Collectively, these findings indicate that ANP exerts multi-targeted effects on neurons, microglia, and the vasculature. Nevertheless, whether ANP modulates angiogenesis in the ischemic brain via skewing microglia toward an M2-like state remains to be elucidated.

This study was designed to evaluate ANP’s neuroprotective effects dependent on post-stroke angiogenesis, investigate its angiogenic promotion through M2-like microglial reprogramming, and map the signaling axes regulating microglia–endothelial interactions.

## 2. Materials and Methods

### 2.1. Animals

C57BL/6J male mice (Sichuan Vital River Laboratory Animal Technology Co., Ltd., Chengdu, Sichuan, China), aged 5–6 weeks with an initial body weight of ~25 g, were used in this study. Animals were housed in a temperature- and humidity-controlled room under a 12 h light-dark cycle with free access to food and water. All experimental procedures were approved by the Experimental Animal Ethics Committee of Chengdu University of Traditional Chinese Medicine on 2 July 2025 (ethical approval number: 2025115). Before the experiment, all animals were housed for at least one week to acclimation to the housing facility.

### 2.2. Cell Culture

The mouse microglial cell line (BV-2) and mouse brain microvascular endothelial cell line (bEnd.3), both purchased from ATCC (Manassas, VA, USA), were routinely cultured in Dulbecco’s modified Eagle’s medium (DMEM; Gibco, (Thermo Fisher Scientific, Waltham, MA, USA)) supplemented with 10% fetal bovine serum (FBS; Gibco) and 1% penicillin–streptomycin. Cells were maintained at 37 °C in a humidified incubator with 5% CO_2_.

### 2.3. Animal Surgery, Ferric Chloride Soak, and ANP Treatment

Mice were randomly assigned (simple randomization) to sham injury (surgery + saline), model (surgery + ferric chloride), or ANP (surgery + ferric chloride + ANP) groups. Animal experimentations and analysis were done blinded to group allocation. For surgery, the method was similar with previous studies [33,34]. Simply, animals were anesthetized by intraperitoneal injection of 3% pentobarbital sodium (2 mL/kg). Vet ointment was applied to the eyes to prevent dryness during anesthesia. The mice were then placed on a heating pad maintained at 37 °C. For thrombus induction, the mice were placed in the supine position and the cervical skin was shaved and sterilized. A 0.8 cm midline neck incision was made to expose the left common carotid artery, beneath which a plastic strip was placed to isolate the surrounding tissues from ferric chloride (FeCl_3_). A piece of Whatman™ filter paper (1 × 8 mm) soaked in freshly prepared 30% FeCl_3_ was applied to the artery for 3 min and then removed. After rinsing with PBS and gentle drying with sterile wipes, the wound was sutured and sterilized (see Supplementary Figure S1 for representative images of the model establishment). The dose of ANP (Product No. 365645, Sigma-Aldrich (St. Louis, MO, USA)) used in this study (2.5 mg/kg) was determined based on previous studies [24,26,35,36] and preliminary experiments. A stock solution of ANP (25 mg/mL in dimethyl sulfoxide, DMSO) was diluted in 200 μL of a long-chain fat emulsion (C8-24Ve) prior to use. The solution was administered to the lesioned animals via daily intraperitoneal injection, starting 7 days before surgery and continuing daily until sacrifice. Sham and model control animals received daily injections of vehicle (DMSO in long-chain fat emulsion without ANP) at equivalent volumes and according to the same schedule (Figure 1A). To minimize potential confounding effects associated with injection order, animals from the three experimental groups were injected in an alternating sequence.

### 2.4. Neurological Function Evaluation

Neurological function was evaluated 4 h post-ischemia using the Zea–Longa scoring system (Table 1) [37]. To ensure experimental consistency and clinical relevance, only mice with mild neurological deficits (scores 1–3) were included in further experiments.

### 2.5. Quantification of Infarct Volume

Infarct volume was quantified by 2,3,5-triphenyltetrazolium chloride (TTC) staining [38]. Coronal brain sections (2 mm thick) were cut 24 h after surgery, incubated in 2% TTC solution (Sigma-Aldrich) at 37 °C in the dark, and postfixed in 4% PFA for 24 h. Infarct areas on individual sections were measured using ImageJ 1.54p. The percentage of infarct volume (%) = (contralateral hemisphere area − (ipsilateral hemisphere area − infarct area))/contralateral hemisphere area × 100%.

### 2.6. Behavioral Assessments

Behavioral performance was assessed using the adhesive tape removal, foot-fault [39], and beam walk tests [40]. Mice were trained for 3 consecutive days before surgery. Baseline measurements were obtained 1 day before surgery (Pre), and follow-up assessments were conducted on postoperative days 3, 5, 7, 14, and 20 (Figure 1A).

In the adhesive tape removal test, a 2 × 3 mm adhesive tape was attached to the contralateral forepaw, and the times to first contact and complete removal were recorded, with a maximum observation period of 120 s. Values were averaged from three trials. For the foot-fault test, mice freely explored an elevated wire grid for 2 min, during which contralateral forelimb placements and foot faults (the forelimb fell through the grid) were counted. Foot-fault rate was expressed as the percentage of foot faults relative to total contralateral forelimb placements. In the beam walking test, an elevated beam (700 mm long, 5 mm wide) led to an enclosed box. Only mice that traversed the beam and entered the box within 20 s without stopping during pre-surgery training were included. The latency to traverse the beam and enter the box was recorded, with a 60 s maximum.

### 2.7. Cerebral Blood Flow Analysis by Laser Speckle Contrast Imaging

The cerebral blood flow (CBF) was measured by Laser Speckle Imaging System (LSCI) RFLSI III (Redward, Shenzhen, China), which emits coherent near-infrared light from a laser diode (785 nm). The measurement procedure was performed as previously publication [41]. Briefly, under anesthesia, the mouse skull was exposed, and blood perfusion rates were recorded at a frame rate of 15 Hz with a resolution of 2048 × 2048 pixels for a 5 s follow-up period. The presurgical blood perfusion rate was established as the baseline, and CBF was recorded on days 10 and 20 after ischemic stroke using the same procedure. To obtain color coding corresponding to CBF velocity (red = high flow, blue = low flow), the pseudo-color threshold range for CBF images was set from 60 to 560. All CBF measurements were conducted within a 1 mm-diameter region of interest (ROI) in the ischemic core of the left temporal lobe. The relative blood perfusion rates were statistically analyzed (*n* = 5).

### 2.8. Immunohistochemistry Staining and Immunofluorescence Staining

Brain tissues were fixed in 4% paraformaldehyde at 4 °C overnight, embedded in paraffin, and sectioned into 5 μm-thick slices. For immunohistochemistry (IHC) staining, endogenous peroxidase activity was quenched with 1.5% (*v*/*v*) H_2_O_2_ in methanol and antigen retrieval was performed by heating the sections in citrate buffer (pH 6.0) at 98 °C for 5–10 min. After antigen unmasking, the sections were incubated overnight at 4 °C with the following primary antibodies: NeuN (1:200; Cell Signaling Technology, (CST; Danvers, MA, USA)), CD31 (1:200; CST), and IBA-1 (1:50; GeneTex (Irvine, CA, USA)). After washing, the sections were incubated with biotinylated secondary antibodies (Vector Laboratories (Newark, CA, USA)) for 1 h at room temperature, followed by ABC reagent (Vector Laboratories) for 30 min. Immunoreactivity was visualized using a 3,3′-diaminobenzidine (DAB) solution. For immunofluorescence (IF) staining, the standard protocol was carried out as previously described [42]. Briefly, slides or cells were incubated with the primary antibodies CD31 (1:200, CST), VEGFR2 (1:100, CST), IL-10 (1:100, SANTA (Dallas, TX, USA)), IBA-1 (1:50, GeneTex), Claudin-5 (1:200, Abcam (Cambridge, UK)) overnight at 4 °C, and then incubated with Alexa 594-conjugated or Alexa 488-conjugated secondary antibodies at room temperature for 1 h. Nuclei were visualized with 4′,6′-diamidino-2-phenylindole (DAPI) staining. Images were captured using a confocal microscope (LSM510, Zeiss (Jena, Germany)). Relative expression level was analyzed using ImageJ based on the integrated optical density (IOD).

### 2.9. Cell Viability Assay

BV-2 or bEnd.3 cells were seeded into 96-well plates. When the cell density reached 60–70%, the cells were treated for 24 h with various concentrations of ANP (1, 5, 10, 15, and 20 μM) in hypoxic nutrient-restricted medium (HNRM; containing 2% FBS and 200 μM CoCl_2_) [35,43]. Cell viability was assessed using the Cell Counting Kit-8 (CCK-8, Absin (Shanghai, China)) according to the manufacturer’s instructions.

### 2.10. Boyden Chamber Migration Assay

BV-2 cells were seeded into 24-well plates and pretreated with or without 1 μM ANP in HNRM for 24 h. bEnd.3 cells were suspended in the same medium and seeded into Transwell inserts (100 μL/well, 8 μm pore size, Cat# 353097, FALCON (Corning Incorporated, Corning, NY, USA)), which were then placed into the corresponding wells containing the pretreated BV-2 cells. After 24 h of co-culture, non-migrating cells on the upper surface of the membrane were removed using a cotton swab. Migrated bEnd.3 cells on the lower membrane surface were visualized using crystal violet staining and manually counted in eight randomly selected microscopic fields under a light microscope.

### 2.11. Matrigel-Based Tube Formation Assay

BV-2 cells were cultured in 6-well plates under HNRM conditions, with or without 1 μM ANP. After pretreatment, BV-2 cells were mixed with bEnd.3 cells at a 1:4 ratio and seeded into Matrigel-coated (BD Biosciences (Franklin Lakes, NJ, USA)) 24-well plates at a total density of 1.2 × 10^6^ cells per well. The co-cultures were incubated for 8–12 h to allow tube formation. Endothelial tube formation was monitored under a microscope and images were captured using an inverted immunofluorescence microscope after calcein AM staining or not. The cumulative tube numbers and tube lengths were quantified using ImageJ software.

### 2.12. Quantitative Real-Time PCR Analysis

Total RNA was extracted from brain tissues, bEnd.3 cells and BV-2 cells using the TRizol Reagent (Thermo Fisher Scientific, Waltham, MA, USA). The RNA was quantified using a spectrophotometer (Denovix (Wilmington, DE, USA)). RNA (1000 ng) was used as a template and was subjected to reverse transcription using an iScript cDNA synthesis kit (Bio-Rad Laboratories, Hercules, CA, USA). Real-time quantitative PCR (RT-qPCR) was performed in duplicate for each sample using a Bio-Rad Real-Time PCR system (CFX96 Optics Module BioRad). Relative gene expression levels were analyzed using the 2^−ΔΔct^ method with an internal control. The primer sequences used in this study are shown in Supplementary Table S1.

### 2.13. Western Blotting

Proteins from left hemisphere brain tissue, BV-2 cells and bEnd.3 cells were extracted using RIPA lysis buffer. The protein concentration was evaluated using a BCA kit (Biosharp (Hefei, China)). Proteins were denatured at 98 °C for 10 min, separated using sodium dodecyl sulfate-polyacrylamide gel electrophoresis (SDS-PAGE), and transferred onto polyvinylidene fluoride (PVDF) membranes. After blocking with 5% fat-free milk, the membranes were incubated overnight at 4 °C with the following antibodies: CD31 (1:1000, CST), VEGFR2 (1:500, CST), IBA-1 (1:500, GeneTex), IL-10 (1:1000, SANTA), ZO-1 (1:1000, GeneTex), Claudin-5 (1:1000, Abcam), VE-cadherin (1:500, CST), CD86 (1:400, GeneTex), and CD206 (1:500, CST). Images were captured using an ImageQuan LAS4000 Image Station (GE Healthcare Bio-Sciences AB, Uppsala, Sweden), and relative expression was quantified using the ImageJ software.

### 2.14. Co-Immunoprecipitation Assay

Total protein was extracted from the whole mouse brain using RIPA buffer. Tissue lysates were precleared using anti-species-specific IgG beads. The precleared tissue lysate was incubated with anti-IL-10 (SANTA) or anti-VEGFR2 (CST) for 1 h at 4 °C. Antibody–antigen complexes were captured by incubation with pre-equilibrated Protein A/G agarose beads overnight at 4 °C on a rocking platform. Co-immunoprecipitated (Co-IP) targets were evaluated using Western blotting.

### 2.15. siRNA Transfection

Scrambled siRNA and siRNA targeting IL-10 were synthesized by GenePharma (Shanghai, China). The siRNAs were transfected into BV-2 cells using Lipofectamine 2000 reagent (Thermo Fisher Scientific, Waltham, MA, USA) according to the manufacturer’s protocol.

### 2.16. Quantification and Statistical Analysis

All data are presented as the mean ± SD. Statistical analyses were performed using Prism 10.0. (GraphPad Software, San Diego, CA, USA). After verifying normality with the Kolmogorov–Smirnov test, a two-tailed unpaired Student’s *t*-test was used for two-group comparisons. For multiple groups, one-way or two-way ANOVA was applied, followed by the Bonferroni post hoc tests. Behavioral data collected at repeating time points were analyzed using two-way repeated measures ANOVA, followed by the Bonferroni post hoc tests * *p* < 0.05 were considered statistically significant.

## 3. Results

### 3.1. ANP Attenuates Ischemic Brain Injury and Functional Deficits

To evaluate the long-term neuroprotective effects of ANP in ischemic stroke, we developed a chronic cerebral ischemia model using FeCl_3_-induced thrombosis in the left common carotid artery of C57BL/6J mice. ANP-treated mice had significantly smaller infarcts than model group (*p* < 0.01) (Figure 1B,C). Moreover, Western blotting of the left hemisphere showed that ischemia markedly reduced NeuN protein levels, and ANP significantly attenuated this reduction at both 10 and 20 dpi (*p* < 0.01) (Figure 1D,E). Immunohistochemistry confirmed that NeuN^+^ immunoreactivity in the peri-infarct cortex (anatomical reference in Figure 1F) was higher in ANP-treated mice at both time points. (*p* < 0.01) (Figure 1F–H). If these structural benefits are meaningful, they should translate into functional improvement. We therefore tested sensorimotor performance using a battery of tests. ANP-treated mice showed shorter contact and removal latencies in the adhesive tape removal test, reduced beam walk crossing latency, and a lower contralateral forepaw fault rate in the foot-fault test (all *p* < 0.05) (Figure 1I–L). These improvements emerged by day 3–7 and persisted through day 20, indicating sustained recovery rather than transient compensation.

The results demonstrate that ANP attenuates acute ischemic brain injury, preserves cortical neurons, and promotes long-term sensorimotor recovery.

### 3.2. ANP Promotes Cerebral Functional Angiogenesis After Ischemic Stroke

ANP has established roles in vascular remodeling [30], endothelial repair [31], gastric homeostasis [32], and BBB protection [29], prompting us to evaluate its potential in post-ischemic vascular function. Therefore, we first investigated the effect of ANP on post-ischemic hemodynamic recovery using LSCI to quantitatively assess CBF dynamics. Longitudinal analysis showed that ischemic hemispheres had sustained hypoperfusion compared with the sham group (*p* < 0.01) (Figure 2A,B), while ANP treatment significantly improved perfusion (*p* < 0.01 vs. model group).

Early increases in cerebral blood volume mainly reflect compensatory collateral circulation, whereas delayed improvements rely on angiogenesis [44]. We thus hypothesized that ANP’s ability to restore CBF may involve enhancing post-ischemic angiogenesis. To test this, we analyzed microvasculature in the cerebral cortex and caudate putamen (CPu) using CD31 immunostaining (anatomical reference in Figure 2C). Quantitative analysis revealed that model mice had significantly shorter total vascular length than the sham group at both 10 and 20 dpi, but systemic ANP administration greatly reduced this vascular loss (Figure 2D–G). Western blotting analysis supported these morphological findings, showing matching changes in CD31 protein expression (Figure 2H–J). To fully evaluate ANP’s impact on vascular integrity, we examined intercellular junction (IJ) proteins, including ZO-1, Claudin-5, and VE-cadherin, in ischemic brains at 10 and 20 dpi. Ischemia significantly depleted these critical junctional components in the model mice compared with the sham group at both time points. Importantly, ANP treatment notably restored their expression (Figure 2H–J).

To gain a comprehensive understanding of how ANP influences post-stroke angiogenesis, RT-qPCR was performed to determine the transcriptional profiles of cerebrovascular genes in the ischemic brains at 10 and 20 dpi. Our findings revealed distinct temporal patterns in vascular gene regulation. In the subacute phase (10 dpi), the model group showed significant upregulation of vascular stability-related genes, including CD31, VEGF, VEGFR2, Tie2, ZO-1, Claudin-5, VE-cadherin, Occludin, and Elastin, compared with the sham group. ANP treatment further strengthened this vascular-stability transcriptional program (Figure 2K). At the chronic phase (20 dpi), however, the model group exhibited paradoxical suppression of most vascular stability-related genes (CD31, VEGF, VEGFR2, Tie2, ZO-1, VE-cadherin and Occludin) relative to the sham group. Here, ANP treatment effectively boosted the expression of these genes (Figure 2L). Strikingly, our temporal analysis revealed a biphasic pattern of angiogenic regulation, in which ischemia triggered compensatory upregulation of vascular stability-related genes during the subacute phase (10 dpi), followed by a decline in the chronic phase. ANP treatment not only enhanced this initial compensatory response but also prevented the later downregulation.

These findings suggest that ANP enhances angiogenesis and preserves endothelial integrity, which together promote long-term vascular stability after ischemia. This functional angiogenesis may provide a foundation for neurological recovery.

### 3.3. ANP Activates M2-Like Microglia Following Ischemic Stroke

Microglia play a key role in regulating angiogenesis and vascular function, particularly through their repair-associated M2-like states [45]. To determine whether ANP modulates microglial responses after ischemia, we first assessed the accumulation of IBA-1^+^ cells using immunohistochemistry. IBA-1^+^ cells were markedly increased in the cortex and caudate–putamen (CPu) of model mice at both 10 and 20 dpi, whereas ANP treatment attenuated these increases (Figure 3A–D). Consistently, Western blot analysis showed elevated IBA-1 protein expression following ischemia, which was reduced by ANP at both time points (Figure 3E–G).

To further characterize microglial/macrophage phenotypic responses, we performed RT-qPCR to examine a panel of M1-like and M2-like state-associated genes in the ischemic hemispheres. At 10 dpi (subacute phase), ischemia upregulated the expression of pro-inflammatory-associated genes (IL-1β, TNF-α, CD86, CD32, IL-15, and C1q) while downregulating that of repair-associated M2-like genes (IL-4, TGF-β, CD206, Ym1, and Arg1), compared with the sham group. ANP partially reversed these transcriptional changes (Figure 3H). A similar effect persisted at 20 dpi (chronic phase), when ANP suppressed M1-like marker expression and increased the expression of several M2-like genes (IL-4, CD206, IL-10, and Arg1) (Figure 3I). Moreover, to validate the transcriptional findings at the protein level, we examined CD86 and CD206 as representative markers of M1-like and M2-like states, respectively, given their widespread use in assessing microglial/macrophage polarization. FeCl_3_-induced ischemia significantly increased CD86 and reduced CD206 protein levels at both time points. Critically, ANP reversed these changes (Figure 3E–G).

In order to assess whether ANP directly induces an M2-like microglial state, BV-2 cells were exposed to ischemia-mimicking hypoxic nutrient-restricted medium (HNRM). HNRM reduced BV-2 cell viability, whereas ANP treatment at concentrations ranging from 1 to 10 μM restored cell viability dose-dependently (Figure 3J); 1 μM ANP was selected for subsequent experiments. LPS treatment (200 ng/mL for 24 h) was used to induce a classical M1-like pro-inflammatory state. As expected, LPS increased the expression of M1-like pro-inflammatory genes (IL-1β, TNF-α, IFN-γ, CD86, and C1q), while decreasing the expression of M2-like genes (IL-4, TGF-β, CD206, IL-10, Ym1, and Arg1) (Figure 3K)—a pattern mirrored in HNRM. ANP suppressed the HNRM-induced pro-inflammatory transcriptional response and restored the expression of M2-like repair-associated genes, supporting a direct effect of ANP on the acquisition of an M2-like microglial state. Notably, ANP amplified IL-10 gene expression beyond ischemia-induced levels (in vivo and in vitro; Figure 3H,I,L), coinciding temporally with vascular-related gene transcription (Figure 2K–L). ANP also reversed the HNRM-induced downregulation of angiogenesis-related genes (VEGF, VEGFR1, and VEGFR2) (Figure 3L).

Collectively, our findings suggest that ANP promotes a potentially pro-angiogenic M2-like microglial state under ischemic conditions.

### 3.4. ANP-Activated M2-Like Microglia Enhance Endothelial Cell Angiogenic Activity

Building on the observation that ANP promotes a potentially pro-angiogenic M2-like microglial state under ischemic conditions, we next investigated the crosstalk between these reprogrammed microglia and endothelial cells. As a prerequisite, we first examined ANP’s cell-type-specific effects under HNRM conditions. Similar to BV-2 cells, bEnd.3 cells exposed to ischemia-mimicking HNRM exhibited comparable ischemic vulnerability, with ANP exerting stable and modest protective effects at 1 μM (Figure 4A), which was selected for subsequent experiments.

To explore the impact of ANP-induced M2-like microglia on endothelial function, we designed a co-culture assay in which BV-2 cells (pretreated with ANP or HNRM) were co-cultured with bEnd.3 cells under HNRM. Tube formation assays (schematic in Figure 4B) showed that HNRM alone severely impaired capillary-like tube formation at 4, 8, and 12 h vs. normoxic controls. Co-culture with HNRM-treated BV-2 cells worsened this suppression (*p* < 0.01, at 8 h). In contrast, ANP-induced M2-like microglia not only restored normal tubulogenesis (Figure 4C,D) but also accelerated the process, inducing tube formation within 2 h, when control endothelial cells showed no tubular structures. Migration assays (Figure 4E–G) showed that HNRM severely impaired bEnd.3 cell migration, with further impairment from a HNRM-pretreated BV-2 co-culture (*p* < 0.01). ANP-pretreated BV-2 cells significantly rescued this deficit. Immunofluorescence for Claudin-5 (Figure 4H–J) confirmed that ANP-activated M2-like microglia preserved endothelial tight junctions against HNRM-induced disruption.

These findings indicate that ANP-activated M2-like microglia promote endothelial cell angiogenic activity under ischemic conditions via enhancing endothelial tubulogenesis, migration, and endothelial integrity.

### 3.5. ANP-Polarized M2-Like Microglia Enhance Endothelial Angiogenic Activity by Secreting IL-10

Previous studies have linked IL-10 to the context-dependent regulation of angiogenesis [46,47]. Our RT-qPCR data further showed that ANP strongly upregulated IL-10 mRNA above the stroke-induced baseline, both in vivo and in vitro (Figure 3H,I,L). To determine if this mRNA increase reflected elevated IL-10 protein in microglia during ischemia, we first assessed IL-10 levels in post-stroke brain tissue. Western blotting showed ischemia alone caused IL-10 significantly increase vs. the sham group, but ANP treatment further amplified this response at both 10 and 20 dpi (*p* < 0.01) (Figure 5A,B). IF staining confirmed the IL-10 colocalized with IBA-1^+^ microglia in ANP-treated ischemic brains (Figure 5C,D). The limited colocalization efficiency likely reflects rapid IL-10 secretion after production, a dynamic needing more investigation.

A direct test of ANP’s effect on microglia under ischemic conditions was performed by culturing BV-2 cells in HNRM and treating them with 1–20 µM ANP for 24 h. Western blotting revealed a dose-dependent rise in IL-10 expression, with 1 μM ANP inducing the strongest response (*p* < 0.01) (Figure 5E,F). Time-course analysis revealed that 1 μM ANP-induced IL-10 upregulation peaked at 12 h post-treatment, then gradually declined, but remained significantly elevated even after 36 h (*p* < 0.01) (Figure 5G,H).

To investigate the IL-10’s role in ANP-mediated angiogenic activity, we used a BV-2 and bEnd.3 cells co-culture system under HNRM conditions. BV-2 cells were received three pretreatments: vehicle, 1 μM ANP, or ANP + IL-10 knockdown (via siRNA, with siRNA-18 most effective; Supplementary Figure S2 and Table S2). Functional assays showed ANP’s pro-angiogenic effects depended critically on microglial IL-10 production. Specifically, IL-10 knockdown in BV-2 cells significantly reduced ANP-induced capillary-like tube formation (*p* < 0.01, Matrigel assay; Figure 6A,B), endothelial migration (*p* < 0.01, Boyden chamber assay; Figure 6C,D), and claudin-5 upregulation (*p* < 0.01, IF staining, Figure 6E,F). In complementary gain-of-function experiments, BV-2 cells were treated with vehicle alone (control), 1 μM ANP, or ANP combined with recombinant IL-10 under HNRM. These showed that IL-10 supplementation further enhanced ANP-induced capillary-like tube formation (*p* < 0.01, Matrigel assay; Figure 6G,H); bEnd.3 migration (*p* < 0.01, Matrigel assay; Figure 6I,J); and Claudin-5 expression (*p* < 0.05, IF staining, Figure 6K,L).

Collectively, these comprehensive findings establish that ANP might induce microglial polarization toward an IL-10-secreting M2-like state following ischemic stroke, which could bridge its angiogenic properties.

### 3.6. ANP-Polarized M2-Like Microglia Upregulate Endothelial VEGFR2 Through IL-10-Mediated Crosstalk

The VEGFR2 signaling pathway is widely recognized as a master regulator of angiogenesis. First, our gene expression analysis revealed that ANP treatment significantly upregulated VEGFR2 mRNA expression in vivo (Figure 2K,L). Importantly, Western blotting showed that ischemic injury alone elevated VEGFR2 protein levels compared with the sham group, and this response was further potentiated by ANP treatment at 10 and 20 dpi (Figure 7A,B). Furthermore, IF staining provided spatial resolution of this effect, demonstrating that ANP specifically enhanced VEGFR2 expression in vascular endothelial cells (Figure 7C,D).

Since ANP upregulated both IL-10 and VEGFR2 expression, we next examined their protein localization via IF staining. Intriguingly, these two proteins were not co-expressed in either BV-2 or bEnd.3 cells. However, when bEnd.3 cells were treated with exogenous recombinant IL-10, we observed a clear colocalization of IL-10 and VEGFR2 (Figure 8A), suggesting a potential interaction between these two proteins. To further test this possibility, we performed Co-IP using mouse brain lysates. The results confirmed that IL-10 and VEGFR2 exist within the same protein complex (Figure 8B), consistent with the colocalization observed in vitro.

To explore the interaction between IL-10 and VEGFR2 under ischemic conditions, we treated bEnd.3 cells in HNRM with 0.2–1.0 ng/mL recombinant IL-10 for 12 h. Western blotting revealed a clear dose-dependent upregulation of VEGFR2, with concentrations ≥ 0.5 ng/mL inducing significant increases (*p* < 0.01 vs. untreated controls; Figure 8C,D). Time-course experiments with 0.5 ng/mL IL-10 revealed dynamic VEGFR2 regulation, with protein levels progressively accumulating at early time points, peaking at 6 h, and then gradually declining—yet, relative to the HNRM group, VEGFR2 levels remained significantly elevated for at least 36 h (*p* < 0.01) (Figure 8E,F). To confirm that microglia-derived IL-10 mediates ANP-induced VEGFR2 upregulation in endothelial cells, we performed complementary loss- and gain-of-function experiments. Silencing IL-10 in BV-2 cells (silencing experiment results were shown in Supplementary Figure S2 and Table S2) via siRNA significantly blocked the ability of ANP-pretreated BV-2 cells to boost VEGFR2 in HNRM-cultured bEnd.3 cells (Figure 8G,H). Conversely, adding 0.5 ng/mL recombinant IL-10 to ANP-pretreated co-cultures further enhanced the VEGFR2 expression of bEnd.3 cells (Figure 8I,J).

These experiments demonstrate that microglial IL-10 might be a potential mediator of ANP’s effects on endothelial VEGFR2 expression under ischemic conditions (Figure 8K).

## 4. Discussion

Our study characterizes the temporal and molecular effects of ANP on angiogenic activity after ischemic stroke. Previous studies have shown that neovascularization begins within 24 h in the peri-infarct region, peaks during the early post-ischemic phase, and may persist for more than 21 days [48]. Building on these observations, we found that sustained low-dose ANP treatment enhanced post-stroke angiogenic capacity and endothelial integrity. This effect involved a coordinated multicellular response characterized by M2-like microglial reprogramming, increased microglial IL-10 production, and IL-10-dependent endothelial VEGFR2 upregulation. These processes were associated with enhanced endothelial migration and tube formation, restoration of tight-junction proteins, and improved cerebral perfusion. Together, our findings suggest that ANP coordinates immunomodulation and vascular repair after ischemic stroke. As ANP was administered exclusively to young male mice beginning 7 days before ischemia, the present results should be interpreted within this experimental context and do not establish its efficacy when initiated after stroke onset or its generalizability to females.

Although the anti-inflammatory and antiviral properties of ANP have been extensively studied, its role in vascular regulation has received attention only recently. Yu et al. [49] reported the dual role of ANP in modulating both inflammation and angiogenesis in diabetic retinopathy. Duan et al. [50] found that ANP suppressed pro-inflammatory cytokines (IL-1β, IL-6, and TNF-α) while enhancing endothelial tube formation and VEGF expression under high-glucose conditions. Evidence from ANP derivatives further supports its vascular effects: sodium 4-andrographolide-19-yldodecanoate promoted endothelial barrier repair through NRP1-dependent VEGFR2/VE-cadherin signaling [51]. In ischemic models, Yen et al. [29] demonstrated that ANP activates the MAPK-Nrf2-HO-1 signaling in brain endothelial cells, and Wang et al. [31] reported endothelial barrier preservation under oxygen-glucose deprivation (OGD) conditions. However, these effects have been demonstrated predominantly in vitro or in studies focused on barrier protection, leaving the role of ANP in post-ischemic angiogenesis in vivo poorly defined. Notably, while high concentrations have been reported to inhibit endothelial migration and tube formation [52,53,54], our study employed a substantially lower dose (1 μM in vitro), which significantly improved bEnd.3 endothelial cell viability. Moreover, BV-2 microglia preconditioned with low-dose ANP enhanced endothelial migration, capillary-like tube formation, and tight-junction protein expression during co-culture (Figure 4). These findings suggest that the vascular effects of ANP are concentration- and context-dependent and that its angiogenic activity under ischemic conditions may be mediated predominantly through microglial reprogramming rather than direct stimulation of endothelial cells.

Initially, the pro-angiogenic role of M2-like macrophages/microglia was extensively studied in the context of tumors, to date, research on the role of M2-like macrophage/microglia in regulating vascular regeneration has garnered increasing attention. For instance, Fantin et al. [55] found that microglia acted as cellular companions during vascular development, positioning themselves at vascular junctions and interacting with endothelial tip cells. Additionally, microglia are known to release growth factors (e.g., VEGF, bFGF, IL-10) [56] and matrix metalloproteinases (MMPs) (e.g., pro-MMP-9) [57] to stimulate vascular sprouting and promote angiogenesis, even without direct contact with endothelial cells.

IL-10 is a pleiotropic cytokine involved in both immunoregulation and context-dependent angiogenesis. IL-10 derived from plasmacytoid dendritic cells promotes vascularization in endometriosis, whereas local IL-10 blockade suppresses this response [46]. The IL-10/JAK1/STAT3 pathway has also been implicated in tumor angiogenesis in endometrial cancer [58], and IL-10 secreted by senescent-like macrophages promotes neovascularization in endplate sclerosis [59]. Our findings extend these observations to ischemic stroke by showing that ANP increased microglial IL-10 expression both in vivo and in vitro (Figure 5). Increased IL-10 expression was accompanied by elevated CD31 expression (Figure 2), suggesting a relationship between the microglial response and vascular remodeling. Loss- and gain-of-function experiments further supported a functional role for IL-10: IL-10 knockdown attenuated ANP-induced endothelial migration, tube formation, and tight-junction protein expression, whereas recombinant IL-10 enhanced these responses (Figure 6). Moreover, within the concentration range tested (0.2–1 ng/mL), recombinant IL-10 progressively increased endothelial VEGFR2 expression (Figure 8C). These findings indicate that microglia-derived IL-10 is an important mediator of the angiogenic activity of ANP. Previous studies have reported both pro- and anti-angiogenic effects of IL-10 across different concentrations and cellular or pathological contexts. For example, 50 ng/mL recombinant IL-10 promoted endothelial tube formation and increased VEGFA, MMP2, and PDGFB expression through STAT3 signaling [59,60], whereas IL-10 concentrations of 10–100 ng/mL reduced HUVEC proliferation and increased endothelial damage, particularly at 100 ng/mL [61,62]. In this study, IL-10 enhanced endothelial angiogenic activity only under hypoxic nutrient-restricted conditions. Whether higher IL-10 concentrations further enhance, saturate, or reverse functional angiogenesis under ischemia remains to be determined.

Additionally, as a potent immunosuppressive cytokine, IL-10 plays a pivotal role in directing macrophage polarization toward the M2 state while simultaneously suppressing pro-inflammatory mediators. This immunomodulatory capacity has been successfully applied in therapeutic strategies for various conditions, including chronic inflammatory diseases, myocardial infarction, and ischemic acute kidney injury [63,64,65]. Zheng et al. [66] similarly reported that M2-like microglia exert neuroprotective effects after cerebral ischemia through anti-inflammatory mediators, including IL-4, IL-10, and TGF-β. In addition to this established paracrine effect, IL-10 may also act in an autocrine manner to reinforce the M2-like microglial state. However, this potential positive-feedback mechanism remains to be verified by examining microglial phenotypic markers following IL-10 silencing or neutralization.

VEGFR2 transactivation serves as a pivotal regulator in the angiogenic signaling cascade, orchestrating essential functions including proliferation, migration, and survival [67]. This tyrosine kinase receptor mediates angiogenesis, the sprouting of new blood vessels from existing vasculature, a process critical for wound healing, embryonic development, and tissue regeneration. Although VEGF-A is its principal ligand, VEGFR2 activity can also be modulated through receptor crosstalk or transactivation involving factors such as NRP1 [51], IL-8 [68] and ROS [69]. Our findings suggest that VEGFR2 participates in the endothelial response to microglia-derived IL-10. Co-immunoprecipitation detected an association between IL-10 and VEGFR2-containing protein complexes (Figure 8B), while IL-10 treatment produced a time-dependent increase in VEGFR2 expression in bEnd.3 cells (Figure 8E, F). Furthermore, IL-10 loss- and gain-of-function altered endothelial migration, tube formation, and Claudin-5 expression (Figure 6). Collectively, these results support the involvement of a microglial IL-10-dependent endothelial VEGFR2-associated signaling mechanism in the vascular effects of ANP.

Nevertheless, our data do not establish IL-10 as a direct ligand for VEGFR2. IL-10 canonically signals through the IL-10R1/IL-10R2 complex [70] and downstream JAK/STAT3 pathway, which is also functional in vascular endothelial cells [60]. IL-10/STAT3 signaling can further promote angiogenic responses by increasing VEGF-A and other pro-angiogenic mediators [47,60]. Thus, microglia-derived IL-10 may engage endothelial IL-10R and subsequently modulate the VEGF-A/VEGFR2 axis or other VEGFR2-associated signaling mechanisms. Further studies using IL-10R blockade, assessment of VEGFR2 phosphorylation, VEGF-A neutralization, and biophysical binding assays will be needed to distinguish these possibilities and define how IL-10 signaling is linked to VEGFR2-dependent endothelial responses. Additionally, BV-2 cells differ from primary microglia in their transcriptional and stimulus-induced responses and may not fully recapitulate microglial behavior in vivo [71,72]. Despite these differences, the consistent directional changes in inflammatory- and repair-associated markers between our in vitro and in vivo experiments support the observed effects of ANP. Further validation in primary microglia would strengthen these findings.

## 5. Conclusions

The present results identified a previously unrecognized role for ANP in post-stroke functional angiogenesis, at least in part, via IL-10-mediated M2-like microglia–endothelial crosstalk involving endothelial VEGFR2 upregulation. These findings connect the established immunomodulatory effects of ANP with its angiogenic capacity and identify microglia–endothelial crosstalk as a potential therapeutic target for post-stroke recovery.

## Figures and Tables

**Figure 1:**
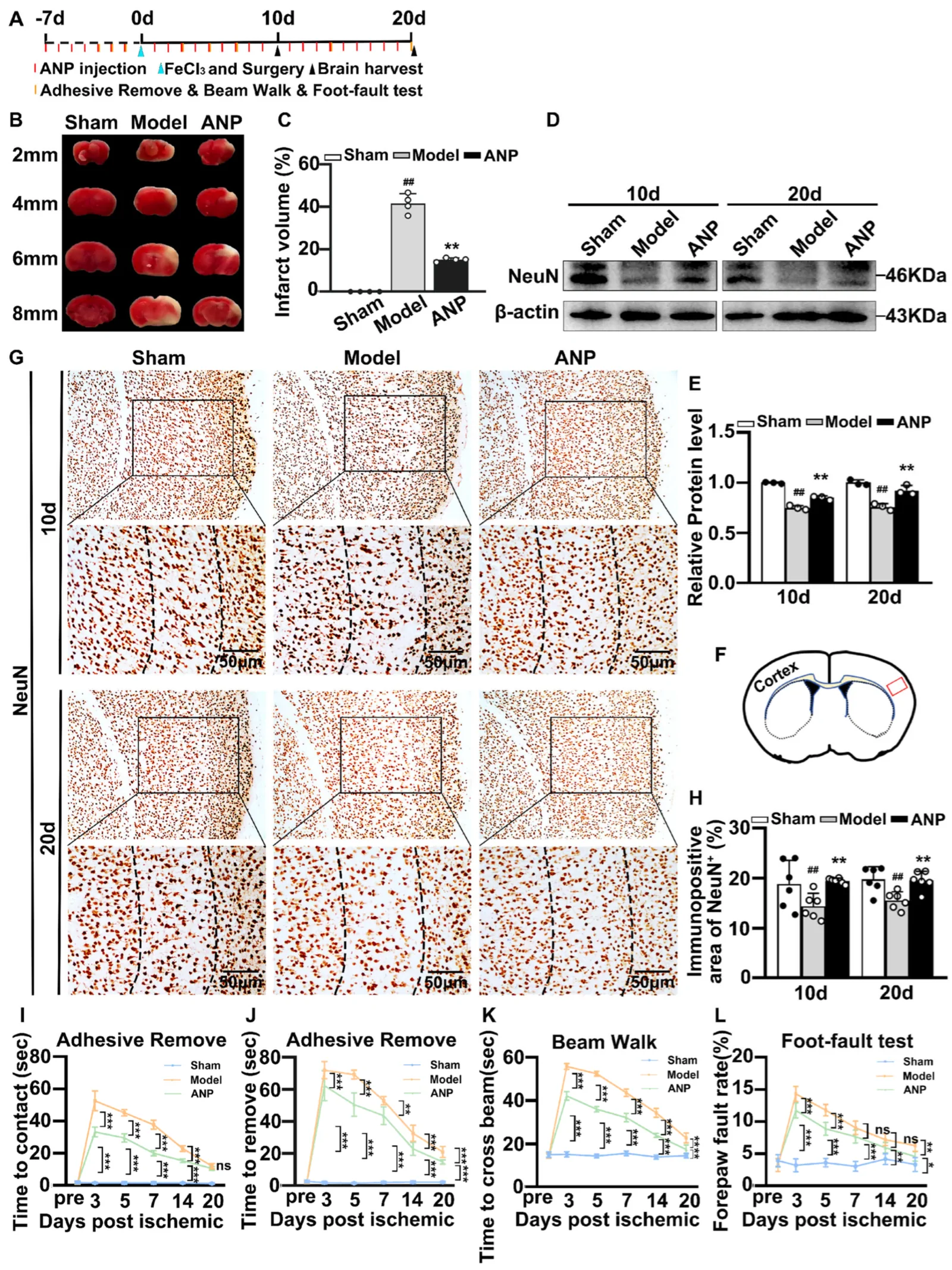
ANP attenuates ischemic brain injury and functional deficits (**A**) Schematic timeline of vehicle or ANP (2.5 mg/kg) treatment, FeCl_3_-induced thrombosis, and behavioral assessments. (**B**,**C**) Infarct volume was determined by TTC staining 24 h after surgery (**B**) representative images; (**C**) quantification, *n* = 4. (**D**–**H**) NeuN protein levels were analyzed by Western blot (**D**) representative blots; (**E**) quantitation, *n* = 3 and by immunohistochemistry (**F**) schematic of anatomical regions; (**G**) representative images; (**H**) quantitation of NeuN^+^ immunoreactivity, *n* = 6 at 10 and 20 dpi. (**I**–**L**) Sensorimotor function was evaluated by adhesive tape removal (**I**) contact latency; (**J**) removal latency), beam walk (**K**), and foot-fault tests (**L**) (*n* = 5 per group for all behavioral tests); using two-way repeated measures ANOVA followed by the Bonferroni post hoc test. Data are presented as means ± SD. Planned comparisons were assessed via one-way ANOVA with linear contrasts (Bonferroni-corrected). # Sham vs. Model, * Model vs. ANP, * *p* < 0.05, ##/** *p* < 0.01, *** *p* < 0.001, ns means not significant. Original uncropped Western blot images are available in Supplementary Figure S3.

**Figure 2:**
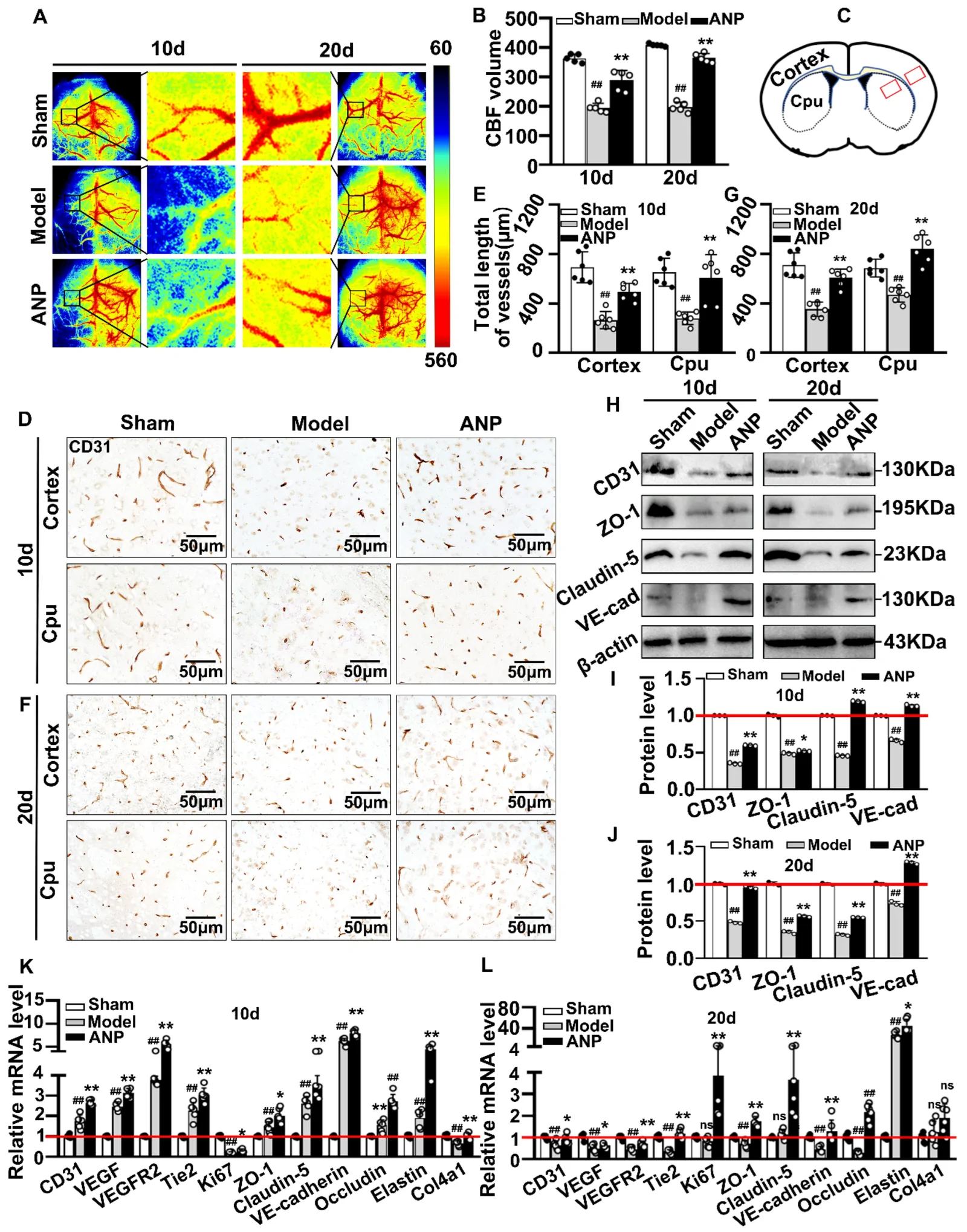
ANP promotes cerebral functional angiogenesis after ischemic stroke (**A**) Representative LSCI of CBF in the left hemisphere at 10 and 20 dpi (**B**) and quantification at each time point, *n* = 5. (**C**–**G**) CD31 immunostaining of microvasculature in the cortex and caudate putamen (CPu) (**C**) schematic of regions of interest; (**D**) representative images at 10 dpi; (**E**) quantification of CD31^+^ vascular length at 10 dpi, *n* = 6; (**F**) representative images at 20 dpi; (**G**) quantification at 20 dpi, *n* = 6. (**H**–**J**) Western blotting of CD31, ZO-1, Claudin-5, and VE-cadherin in the left hemisphere (**H**) representative blots; (**I**) quantitation at 10 dpi, *n* = 3; (**J**) quantitation at 20 dpi, *n* = 3. (**K**–**L**) RT-qPCR analysis of angiogenesis-related genes, including CD31, VEGF, VEGFR2, Tie2, Ki67, ZO-1, Claudin-5, VE-cadherin, Occludin, Elastin, and Col4a1, in the left hemisphere at 10 dpi (**K**) *n* = 6 and 20 dpi (**L**) *n* = 6. Data are presented as means ± SD. Planned comparisons were assessed via one-way ANOVA with linear contrasts (Bonferroni-corrected). # Sham vs. Mode, * Model vs. ANP, * *p* < 0.05, ##/** *p* < 0.01, ns means not significant. Original uncropped Western blot images are available in the Supplementary Figure S4.

**Figure 3:**
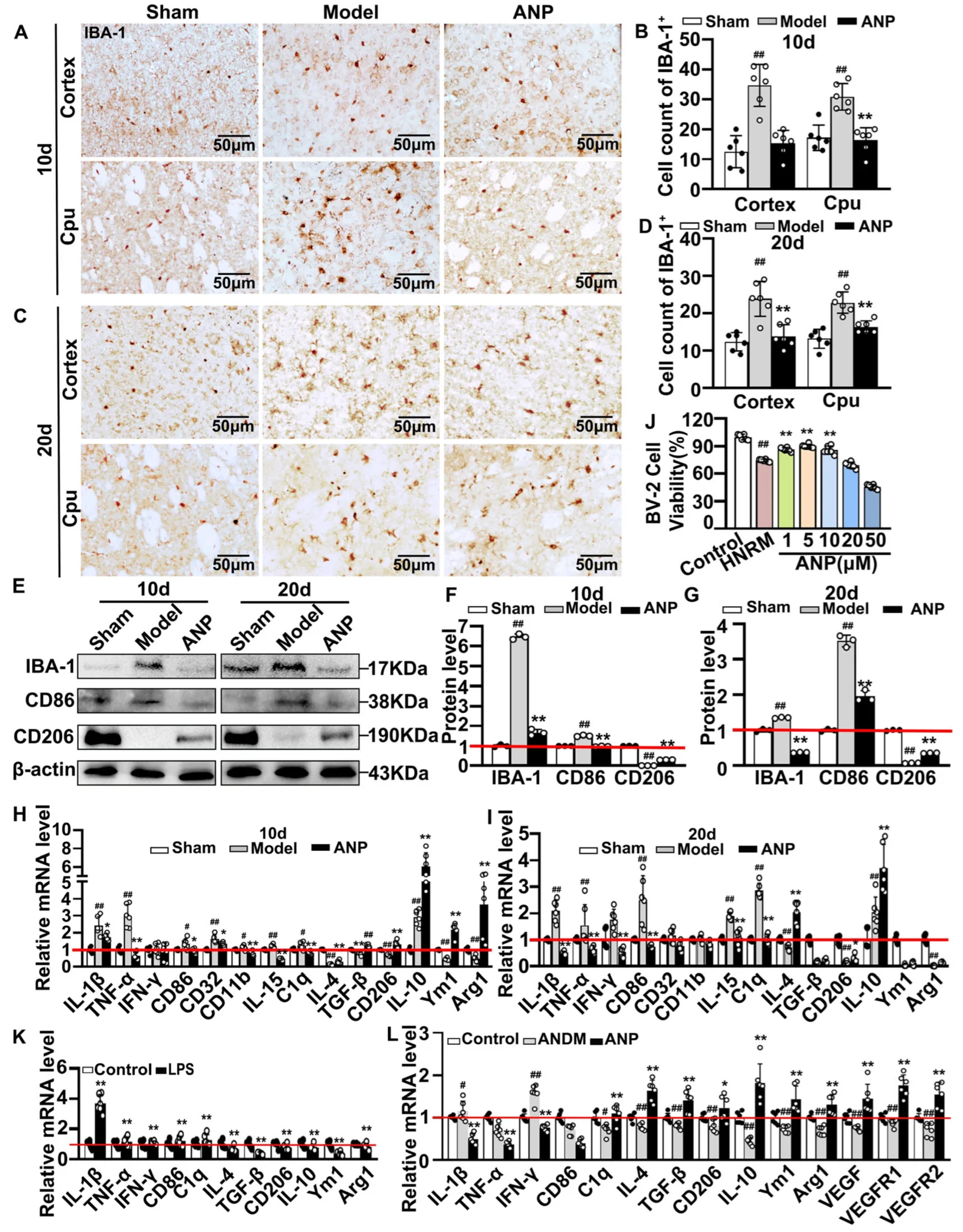
ANP activates M2-like microglia following ischemic stroke (**A**–**D**) IBA-1 immunostaining in the cortex and caudate putamen (CPu) at 10 dpi (**A**) representative images; (**B**) quantification of IBA-1^+^ cells, *n* = 6 and 20 dpi (**C**) representative images; (**D**) quantification, *n* = 6. (**E**–**G**) Western blotting of IBA-1, CD86, and CD206 in the left hemisphere at 10 and 20 dpi (**E**) representative blots; (**F**) quantitation at 10 dpi, *n* = 3; (**G**) quantitation at 20 dpi, *n* = 3. (**H**,**I**) RT-qPCR analysis of M1-associated (IL-1β, TNF-α, IFN-γ, CD86, CD32, CD11b, IL-15, and C1q) and M2-associated (IL-4, TGF-β, CD206, IL-10, Ym1, and Arg1) genes in the left hemisphere at 10 dpi (**H**) *n* = 6 and 20 dpi (**I**) *n* = 6. (**J**) Viability of BV-2 cells treated with various concentrations of ANP for 24 h, *n* = 6; data are mean ± SD; two-tailed unpaired Student’s *t*-test. (**K**) RT-qPCR analysis of M1- and M2-associated markers in BV-2 cells following LPS stimulation (200 ng/mL, 24 h), *n* = 6. (**L**) RT-qPCR analysis of M1- and M2-associated markers in BV-2 cells treated with 1 μM ANP for 24 h under hypoxic nutrient-restricted medium (HNRM) conditions, *n* = 6. Data are presented as means ± SD. Planned comparisons were assessed via one-way ANOVA with linear contrasts (Bonferroni-corrected). # Sham vs. Model/Control vs. HNRM, * Model vs. ANP/HNRM vs. ANP, #/* *p* < 0.05, ##/** *p* < 0.01. Original uncropped Western blot images are available in the Supplementary Figure S5.

**Figure 4:**
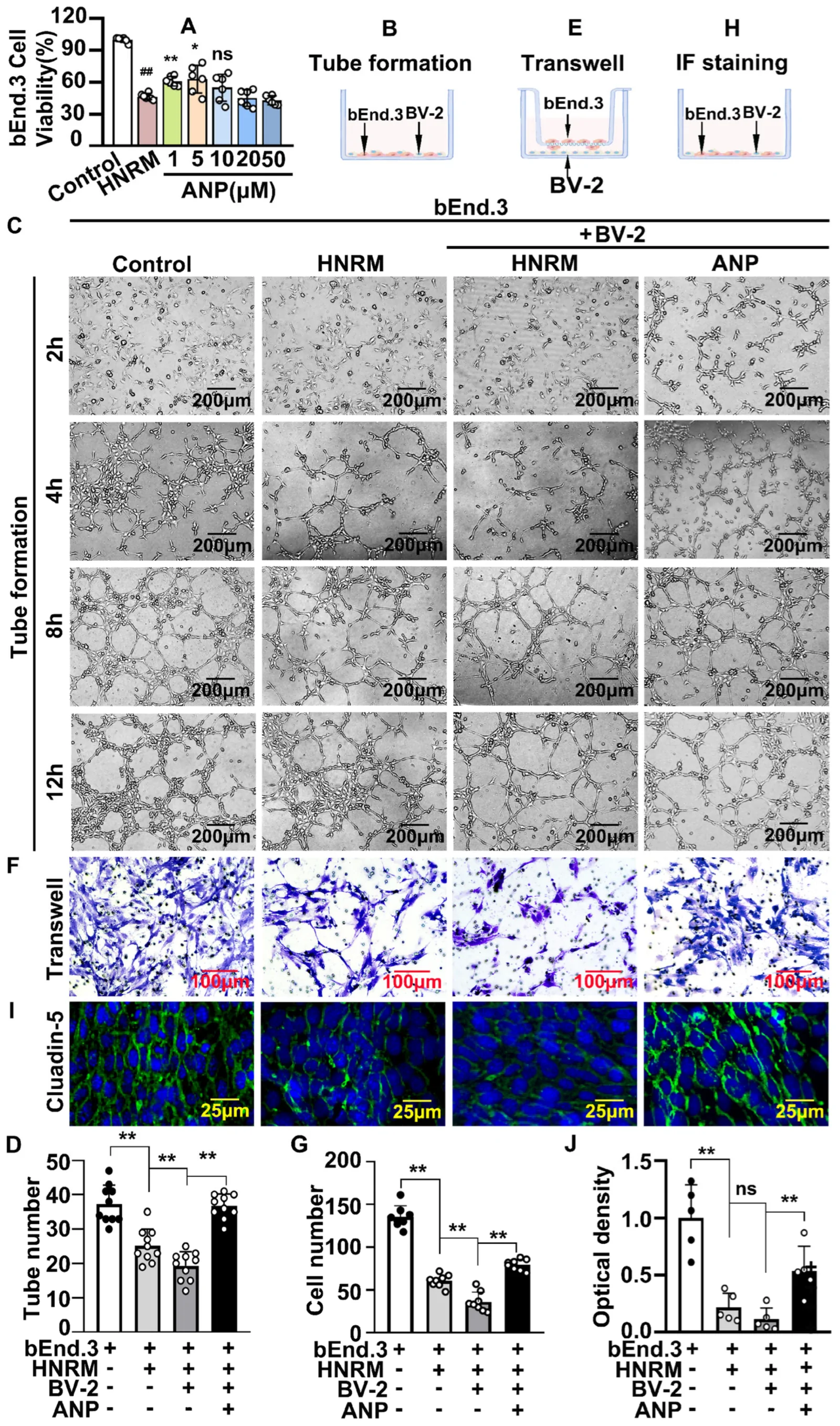
ANP-activated M2-like microglia enhance endothelial cell angiogenic activity (**A**) viability of bEnd.3 cells treated with ANP for 24 h under HNRM conditions, *n* = 6. (**B**–**J**) BV-2/bEnd.3 co-culture system under HNRM conditions with or without 1 μM ANP preconditioning: (**B**) schematic of the Matrigel-based tube formation assay; (**C**) representative images of capillary-like structures at indicated time points; (**D**) quantification of tube numbers at 8 h, *n* = 10; (**E**) schematic of the migration assay; (**F**) representative images of migrated bEnd.3 cells; (**G**) quantification of migrated cells, *n* = 8; (**H**) schematic of the Claudin-5 staining assay; (**I**) representative images of Claudin-5 expression (green) in bEnd.3 cells; (**J**) quantification of Claudin-5^+^ immunoreactivity, *n* = 5. Data are presented as means ± SD. Planned comparisons were assessed via one-way ANOVA with linear contrasts (Bonferroni-corrected). # Control vs. HNRM, * HNRM vs. ANP, * *p* < 0.05, ##/** *p* < 0.01, ns means not significant.

**Figure 5:**
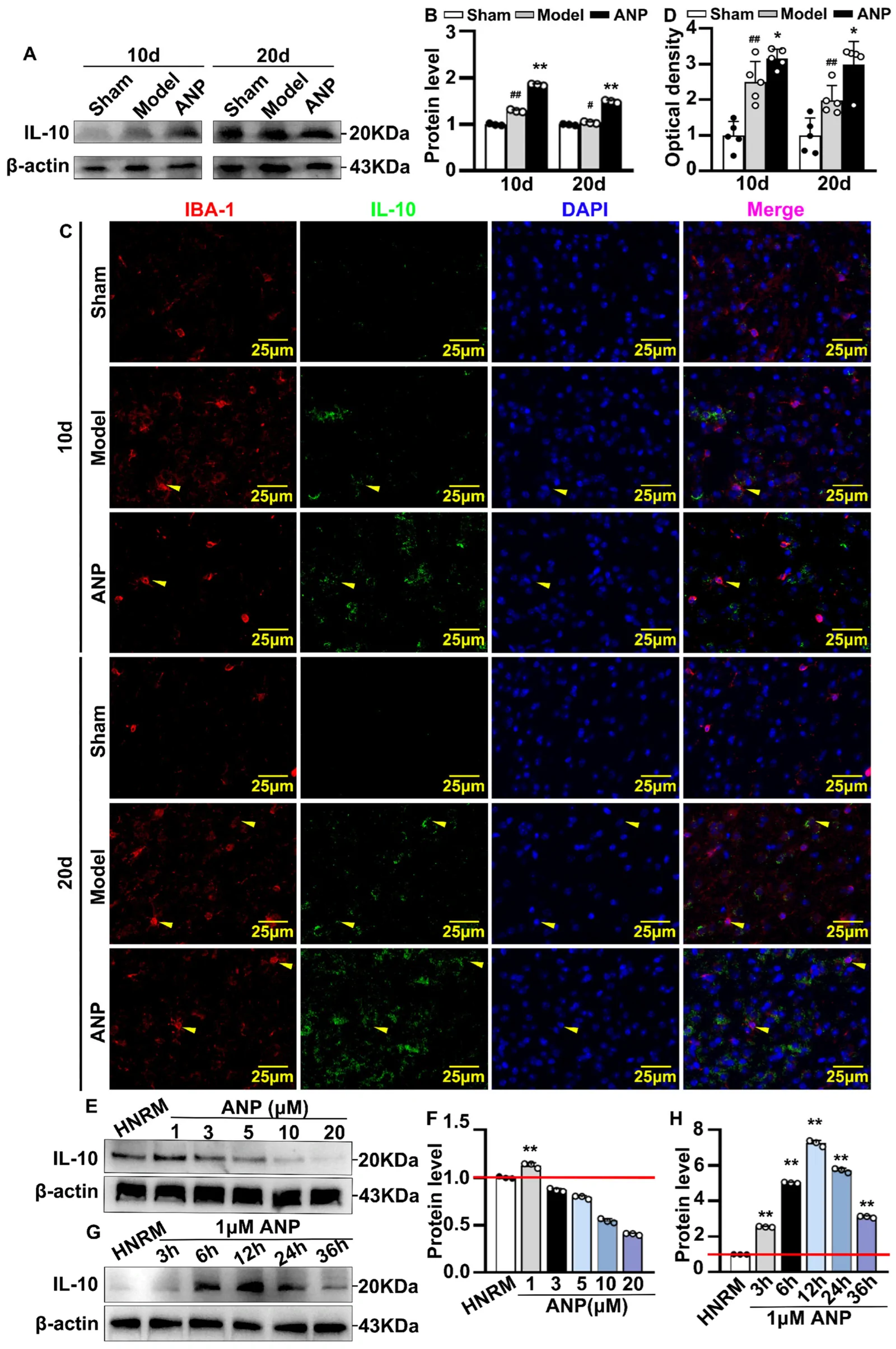
ANP-polarized M2-like microglia enhance endothelial angiogenic activities by secreting IL-10 (**A**–**D**) IL-10 expression in the left hemisphere at 10 and 20 dpi (**A**) representative Western blots; (**B**) quantification of IL-10 protein levels, *n* = 3; (**C**) representative IF images showing IL-10 (green) co-stained with IBA-1 (red) in coronal brain sections; (**D**) quantification of IL-10^+^ immunoreactivity, *n* = 5. (**E**,**F**) IL-10 expression in BV-2 cells exposed to increasing ANP concentrations under HNRM conditions for 12 h (**E**) representative Western blots; (**F**) quantification, *n* = 3. (**G**,**H**) Time-course of IL-10 expression in BV-2 cells treated with 1 μM ANP under HNRM conditions (**G**) representative Western blots; (**H**) quantification, *n* = 3. Data are presented as means ± SD. Planned comparisons were assessed via one-way ANOVA with linear contrasts (Bonferroni-corrected). # Sham vs Model group, * Model vs. ANP group/HNRM vs. ANP, #/* *p* < 0.05, ##/**p < 0.01. Original uncropped Western blot images are available in the Supplementary Figures S6–S8.

**Figure 6:**
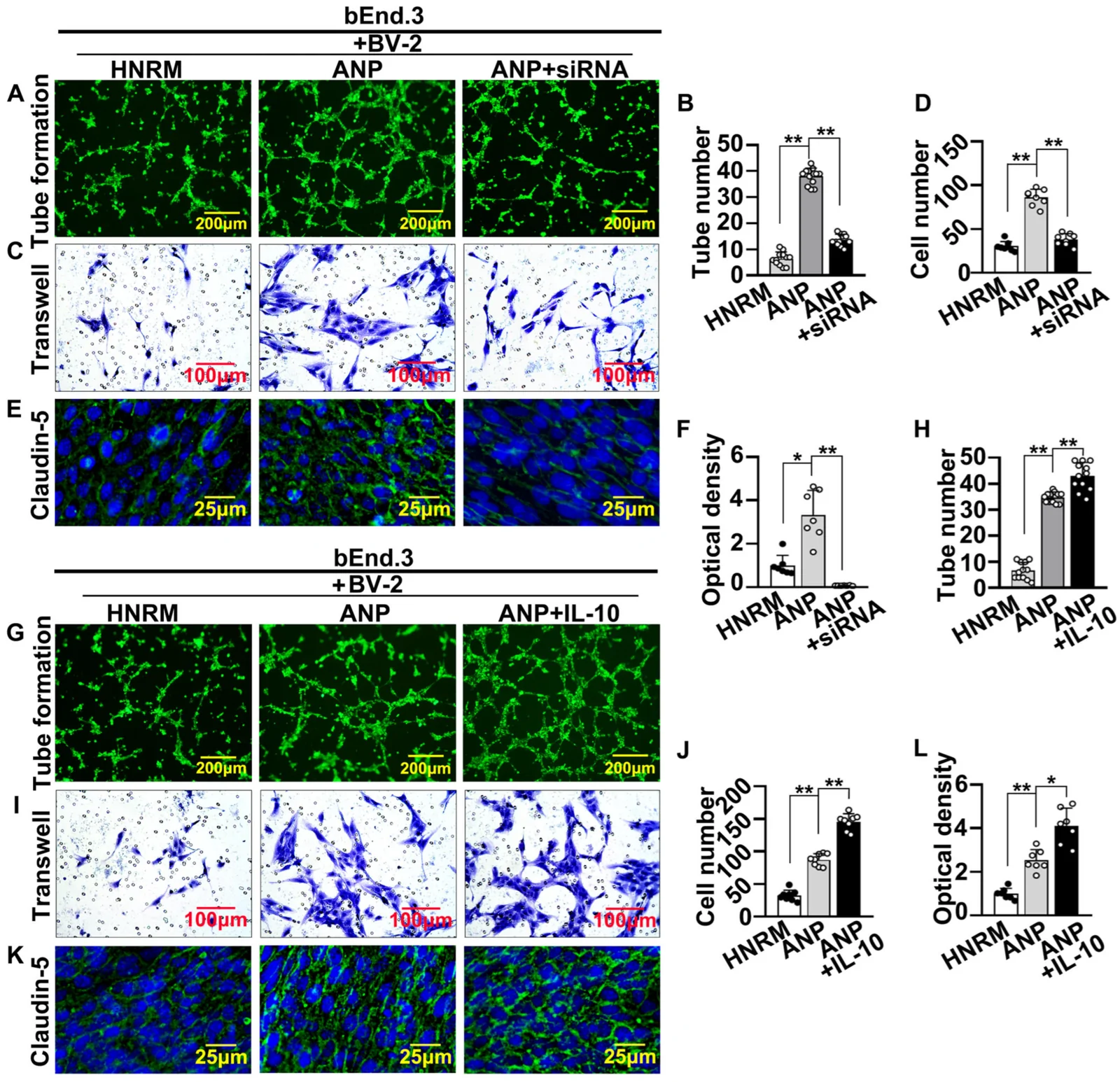
IL-10 secreted from ANP-induced M2-like microglia enhances endothelial angiogenic activity (**A**–**F**) BV-2/bEnd.3 co-culture under HNRM conditions with or without 1 μM ANP preconditioning, with additional si-IL-10 knockdown (**A**) representative images of capillary-like structures at 8 h; (**B**) quantification of tube numbers, *n* = 12; (**C**) representative images of migrated bEnd.3 cells in Boyden chamber assay; (**D**) quantification of migrated cells, *n* = 8; (**E**) representative images of Claudin-5 expression (green) in bEnd.3 cells; (**F**) quantification of Claudin-5^+^ immunoreactivity, *n* = 7. (**G**–**L**) BV-2/bEnd.3 co-culture under HNRM conditions with or without 1 μM ANP preconditioning, with additional 0.5 ng/mL exogenous IL-10: (**G**) representative images of capillary-like structures at 8 h; (**H**) quantification of tube numbers, *n* = 12; (**I**) representative images of migrated bEnd.3 cells in Boyden chamber assay; (**J**) quantification of migrated cells, *n* = 8; (**K**) representative images of Claudin-5 expression (green) in bEnd.3 cells; (**L**) quantification of Claudin-5^+^ immunoreactivity, *n* = 7. Data are presented as means ± SD. Planned comparisons were assessed via one-way ANOVA with linear contrasts (Bonferroni-corrected). HNRM vs. ANP, ANP vs. ANP + siRNA/IL-10, * *p* < 0.05, ** *p* < 0.01.

**Figure 7:**
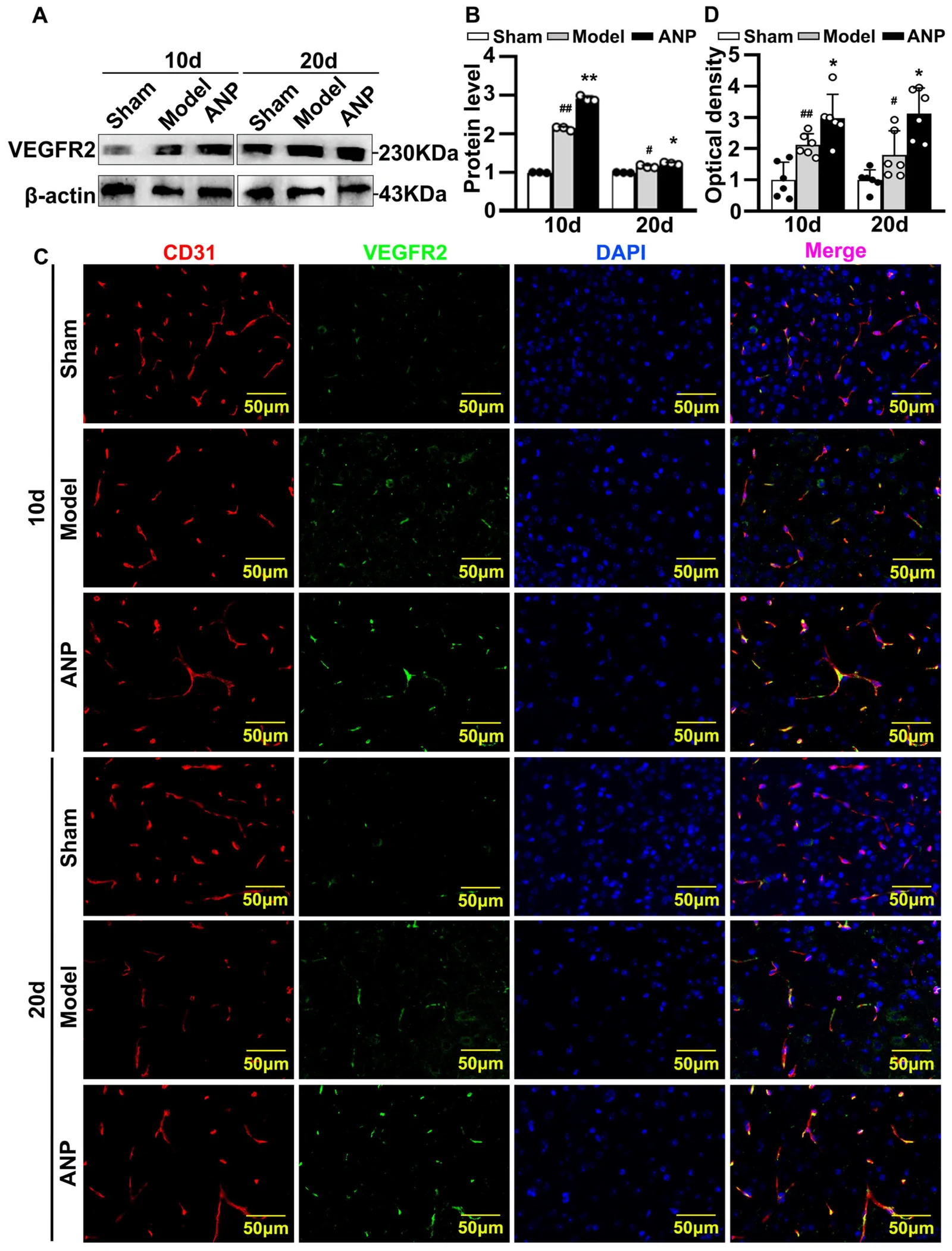
ANP promotes VEGFR2 expression after ischemic stroke (**A**–**D**) VEGFR2 expression in the left hemisphere at 10 and 20 dpi (**A**) representative Western blots; (**B**) quantification of VEGFR2 protein levels, *n* = 3; (**C**) representative IF co-staining of VEGFR2 (green) and CD31 (red) in the cortex; (**D**) quantification of VEGFR2^+^ immunoreactivity, *n* = 6. Data are presented as means ± SD. Planned comparisons were assessed via one-way ANOVA with linear contrasts (Bonferroni-corrected). # Sham vs. Model group, * Model vs. ANP group, #/* *p* < 0.05, ##/** *p* < 0.01. Original uncropped Western blot images are available in the Supplementary Figure S9.

**Figure 8:**
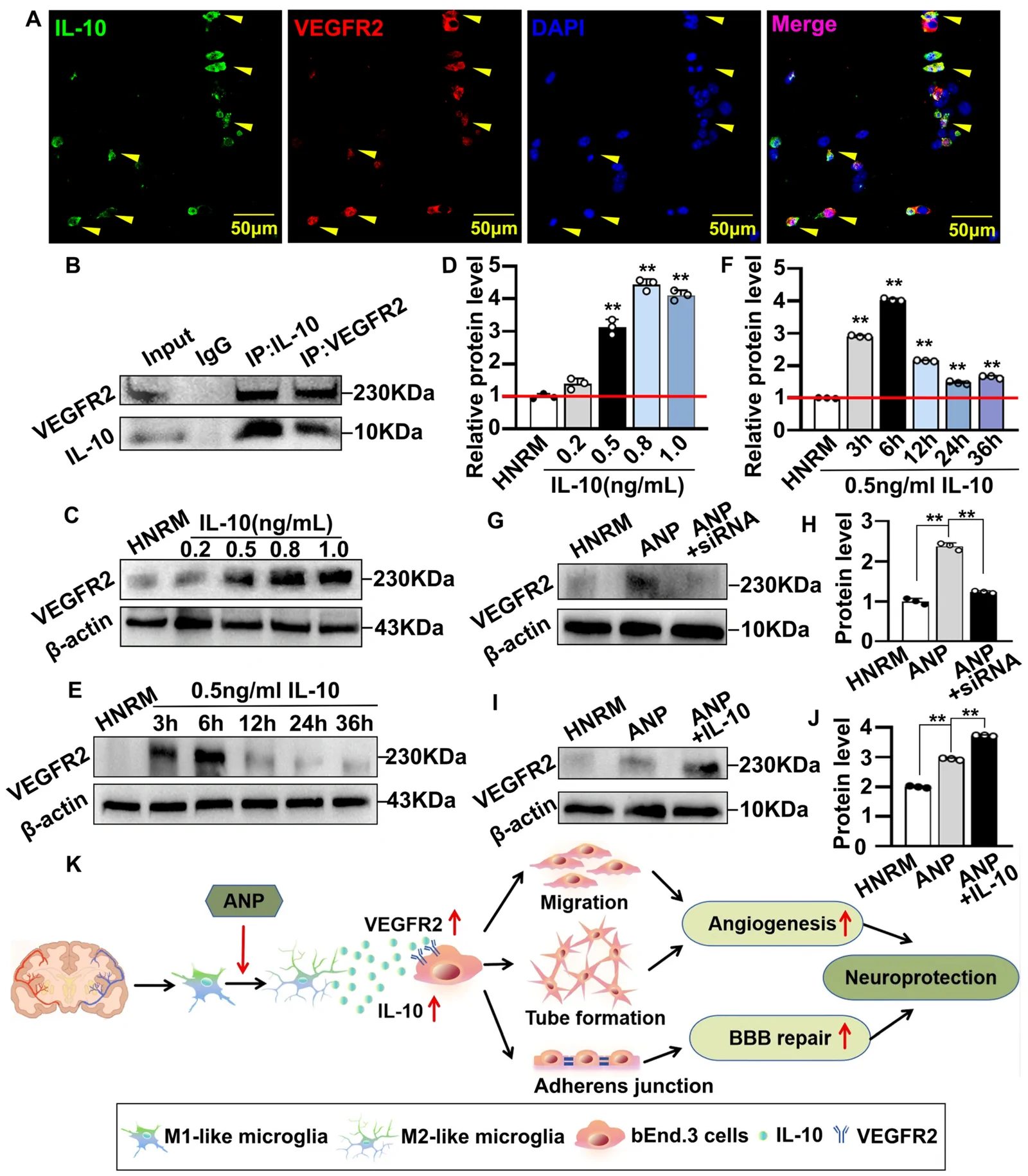
ANP-polarized M2-like microglia upregulate endothelial VEGFR2 through IL-10-mediated crosstalk (**A**–**J**) IL-10 and VEGFR2 interaction (**A**) IF co-staining of IL-10 (green) and VEGFR2 (red) in bEnd.3 cells after treatment with 0.5 ng/mL IL-10 for 6 h under HNRM conditions; (**B**) Co-IP of IL-10 and VEGFR2 from normal mouse brain confirming their physical interaction; (**C**,**D**) VEGFR2 expression in bEnd.3 cells treated with increasing concentrations of recombinant IL-10 under HNRM conditions for 6 h (**C**) representative Western blots; (**D**) quantification, *n* = 3; (**E**,**F**) Time course of VEGFR2 expression in bEnd.3 cells exposed to 0.5 ng/mL recombinant IL-10 under HNRM conditions (**E**) representative Western blots; (**F**) quantification, *n* = 3; (**G**,**H**) VEGFR2 expression in bEnd.3 cells co-cultured with BV-2 cells pretreated with 1 μM ANP or ANP + si-IL-10 under HNRM conditions (**G**) representative Western blots; (**H**) quantification, *n* = 3; (**I**,**J**) VEGFR2 expression in bEnd.3 cells co-cultured with BV-2 cells pretreated with 1 μM ANP under HNRM conditions, with exogenous IL-10 (0.5 ng/mL) added to the co-culture system (**I**), representative Western blots; (**J**) quantification, *n* = 3. (**K**) Schematic summary depicting that ANP promotes M2-like microglial polarization, which secretes IL-10 to activate VEGFR2 signaling in endothelial cells via paracrine pathway, thereby enhancing angiogenesis (migration, tube formation) and functional recovery after ischemic stroke. Data are presented as means ± SD. Planned comparisons were assessed via one-way ANOVA with linear contrasts (Bonferroni-corrected). HNRM vs. IL-10, HNRM vs. ANP, ANP vs. ANP + siRNA/IL-10, ** *p* < 0.01. Original uncropped Western blot images are available in Supplementary Figures S10–S13.

**Table 1:** Neurological function score in mice.

Behavioral Manifestation	Degree of Nerve Injury	Functional Score
No observable deficits	No injury	0
Contralateral forelimb flexion when lifted by tail	Mild injury	1
Circling toward the contralateral side during crawling	Moderate injury	2
Falling to the contralateral side when standing or crawling	Severe injury	3
No spontaneous movement with impaired consciousness	Severe injury	4

## Data Availability

Data will be made available on request.

## Supplementary Materials

The following supporting information can be downloaded at: https://www.mdpi.com/article/10.3390/biology15191715/s1, Figure S1: FeCl_3_ induced carotid artery thrombosis to build an ischemic stroke mouse model; Figure S2: Knockdown efficiency of IL-10 specific siRNA transfection into BV-2 cells; Figure S3: Western blot membrane for Figure 1D; Figure S4: Western blot membrane for Figure 2H; Figure S5: Western blot membrane for Figure 3E; Figure S6: Western blot membrane for Figure 5A; Figure S7: Western blot membrane for Figure 5E; Figure S8: Western blot membrane for Figure 5G; Figure S9: Western blot membrane for Figure 7A; Figure S10: Western blot membrane for Figure 8B; Figure S11: Western blot membrane for Figure 8C; Figure S12: Western blot membrane for Figure 8E; Figure S13: Western blot membrane for Figure 8G,I; Figure S14: Western blot membrane for Figure S2. Table S1: List of primer sequences used for Real Time PCR in the study; Table S2: The specific RNA sequece of siRNA.

## Abbreviations

The following abbreviations are used in this manuscript:

HNRM	hypoxic nutrient-restricted medium
ANP	Andrographolide
bEnd.3	Brain microvascular endothelial cells
BV-2	Microglial cell line
CBF	Cerebral blood flow
CPu	Caudate putamen
dpi	days after ischemia
LPS	Lipopolysaccharide
LSCI	Laser Speckle Imaging System
