# Supplementary material for: Andrographolide Promotes Post-Ischemic Endothelial Angiogenic Activity via IL-10-Mediated M2-Like Microglia–Endothelial Crosstalk Involving Endothelial VEGFR2 Upregulation
Source: Biology (Basel). 2026 Sep 26;15(19):1715. doi: 10.3390/biology15191715 (PMC13663447; doi:10.3390/biology15191715)
Supplement: Supplementary file 1 [file biology-15-01715-s001.zip › biology-4558998-supplementary.pdf]

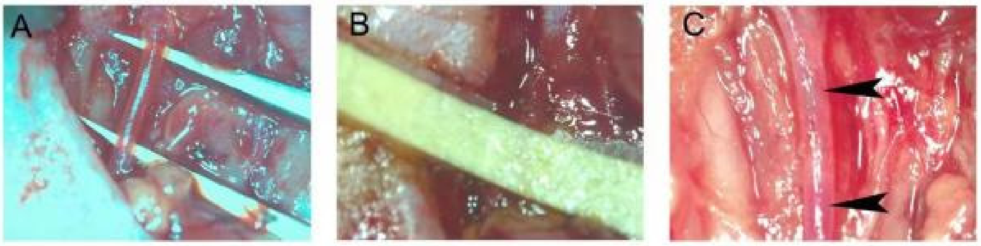

Figure S1. $FeCl_3$ induced carotid artery thrombosis to build an ischemic stroke mouse model (A) A 0.8-cm wound along the midline of the mouse's neck to isolate the left common carotid artery, (B) and then a small white plastic piece was placed the under the isolated part of the common carotid to prevent ferric chloride from soaking into the surrounding tissues. Subsequently, a piece of WhatmanTM filer paper strip (1 mm×2 mm) soaked in freshly prepared 30% ferric chloride was placed on the left common carotid artery. (C) After 3 min exposure, a visible thrombus has formed in the left common carotid artery (indicated by arrow).

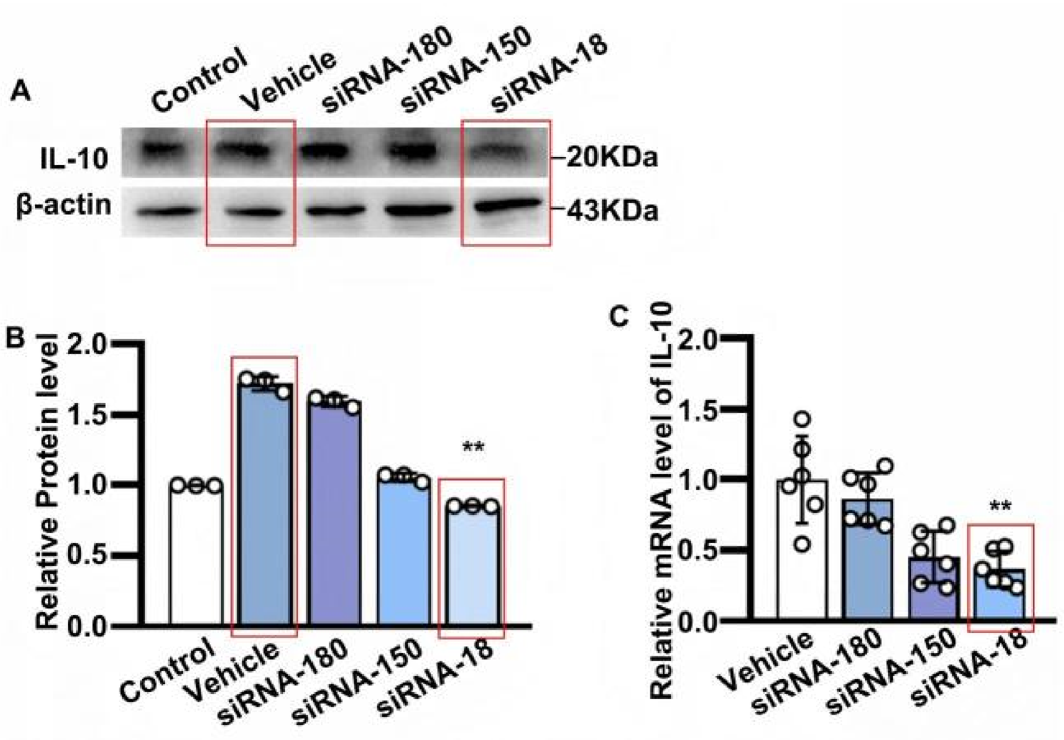

Figure S2. Knockdown efficiency of IL-10 specific siRNA transfection into BV-2 cells (A) IL-10 knock down efficiency tested by WB after BV-2 administrated with siIL-10 for 6-8 h. (B) Quantitative analysis of band intensity in (A). (C) IL-10 knockdown efficiency tested by RT-qPCR after BV-2 administrated with siIL-10 for 6-8 h. *Vehicle vs 18-siRNA group, *$P < 0.01$. Data = mean ±SD.

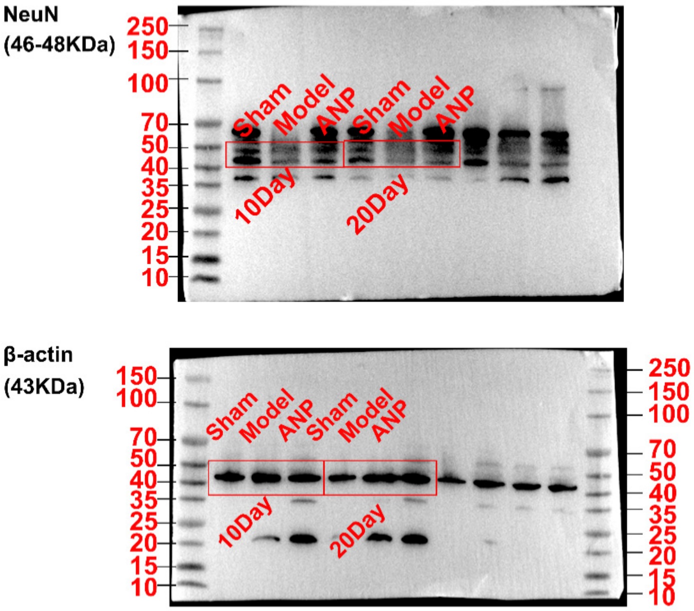

Figure S3. Western blot membrane for Figure 1D.

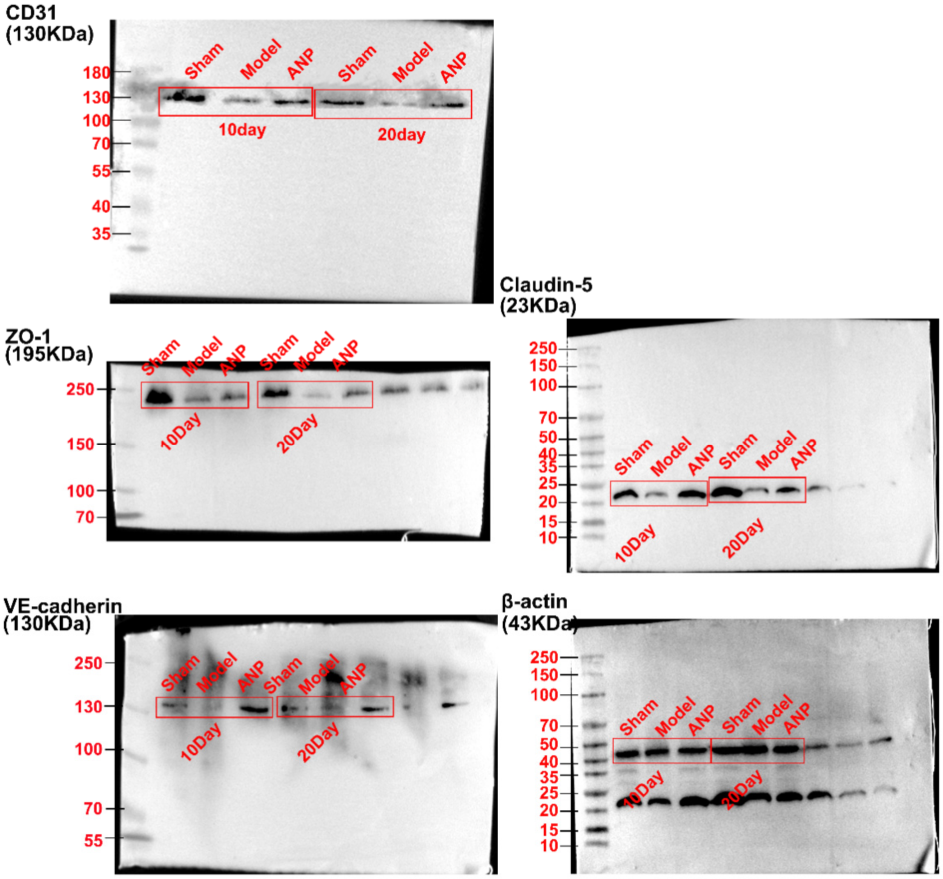

Figure S4. Western blot membrane for Figure 2H.

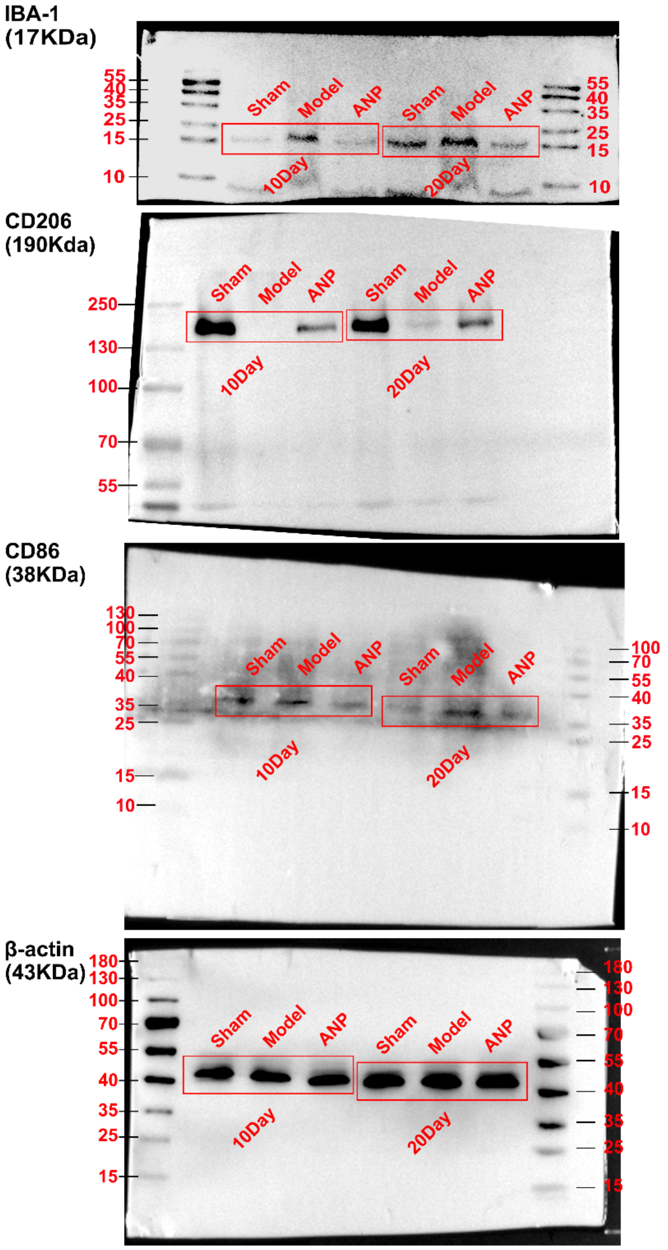

Figure S5. Western blot membrane for Figure 3E.

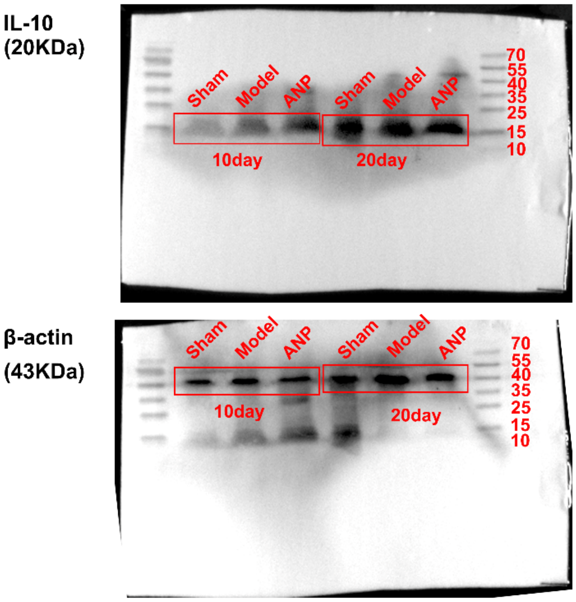

Figure S6. Western blot membrane for Figure 5A.

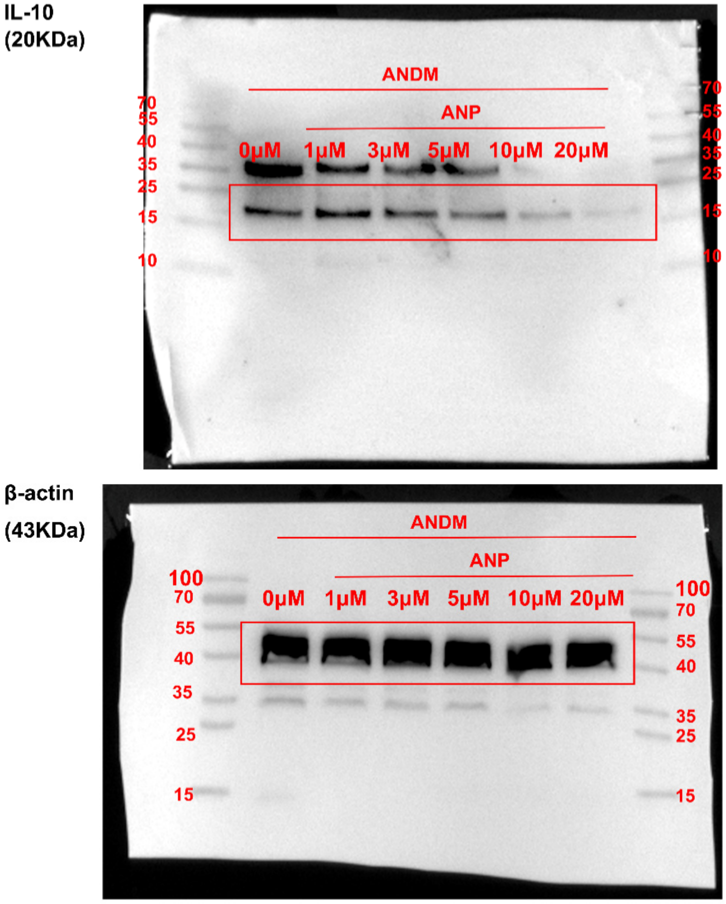

Figure S7. Western blot membrane for Figure 5E.

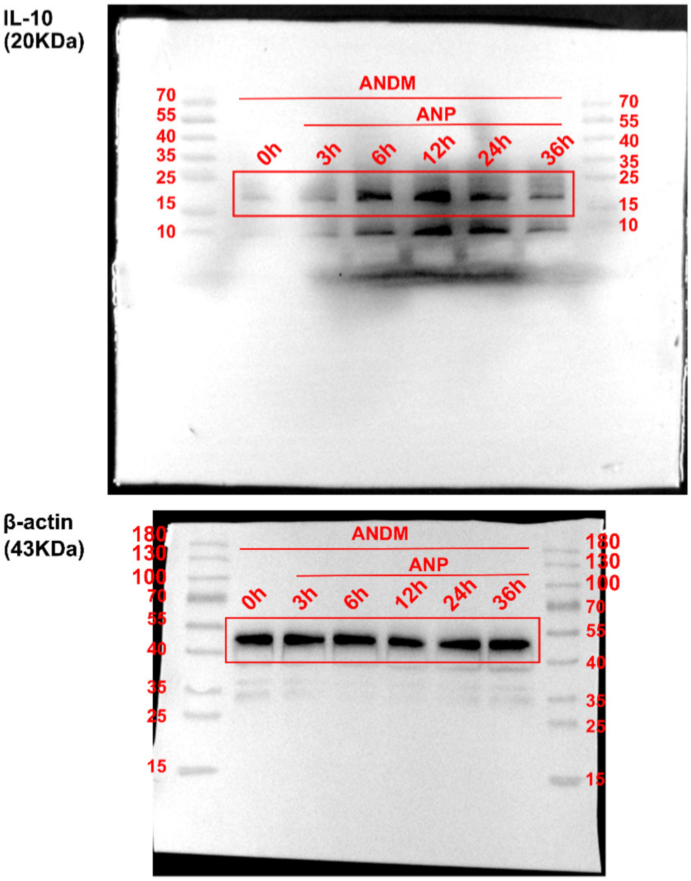

Figure S8. Western blot membrane for Figure 5G.

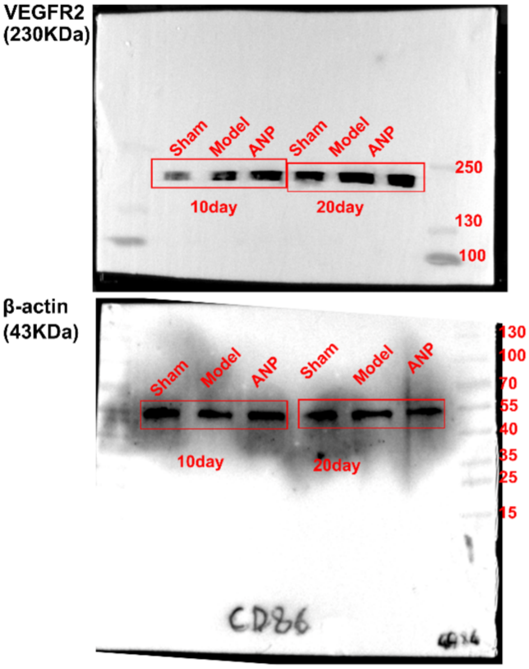

Figure S9. Western blot membrane for Figure 7A.

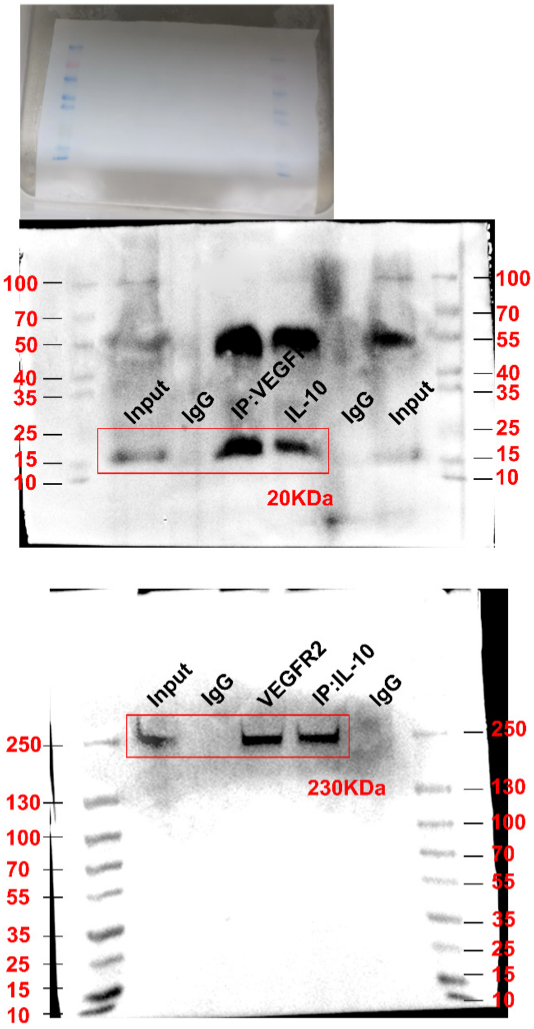

Figure S10. Western blot membrane for Figure 8B.

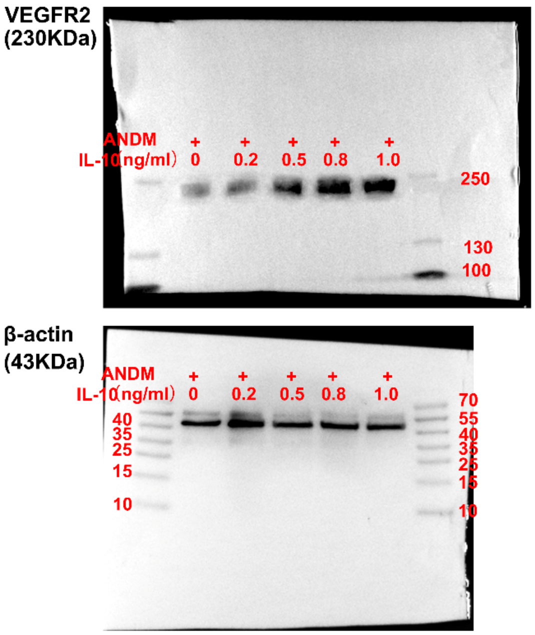

Figure S11. Western blot membrane for Figure 8C.

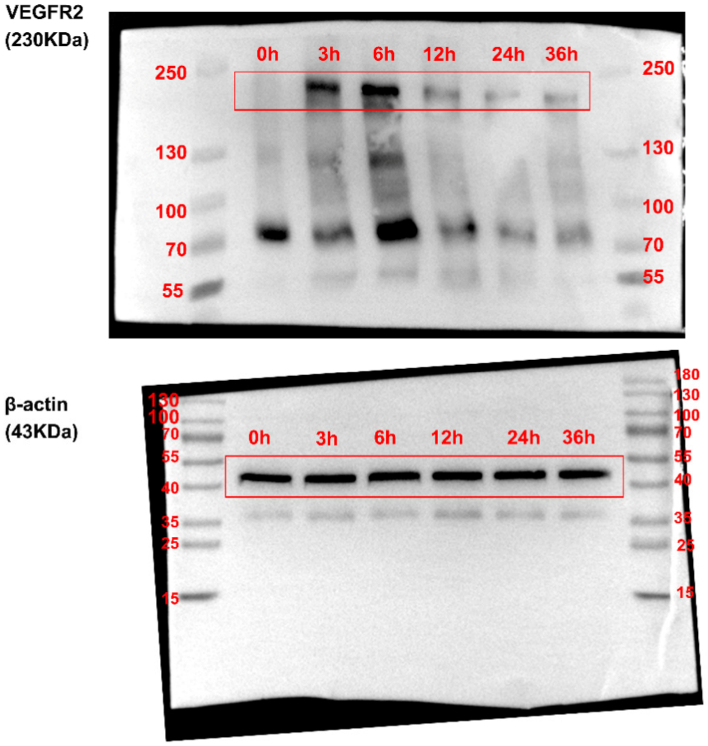

Figure S12. Western blot membrane for Figure 8E.

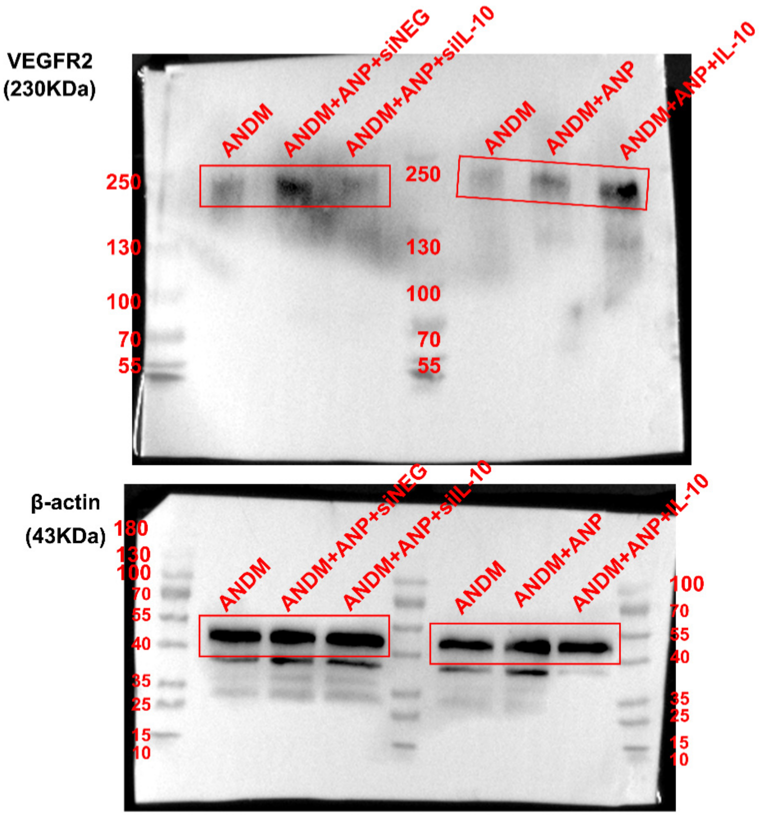

Figure S13. Western blot membrane for Figures 8G and 8I.

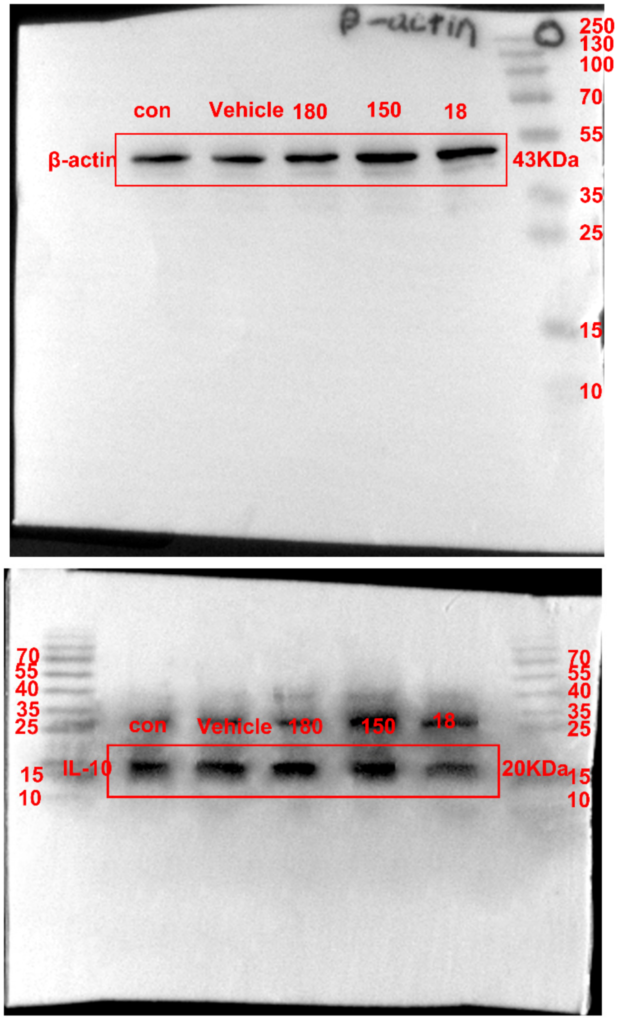

Figure S14. Western blot membrane for Figure S2.

Table S1. List of primer sequences used for Real Time PCR in the study.

Gene Name	Species	Sequence
IL-1β	Mouse	F:5'-GAAATGCCACCTTTTGACAGTG-3′<br>R:5' -TGGATGCTCTCATCAGGACAG-3′
TNF-α	Mouse	F:5' -GATCGGTCCCCAAAGGGATG-3′<br>R:5' -TGGTTTGTGAGTGTGAGGGT-3′
IFN-γ	Mouse	F:5' -AGCAAGGCGAAAAAGGATGC-3′<br>R:5' -TCATTGAATGCTTGGCGCTG-3′
C1q	Mouse	F:5' -GGATGGGGCTCCAGGAAATC-3′<br>R:5' -GATACCAGTCCGGATGCCAG-3′
CD86	Mouse	F:5' -CTTACGGAAGCACCCACGAT-3′<br>R:5' -TGTAAATGGGCACGGCAGAT-3′
CD32	Mouse	F:5' -TGTGCCATATGCTACTGTGGA-3′

Gene Name	Species	Sequence
		R:5' -TCACCGTGTCTTCCTTGAGC-3′
CD11b	Mouse	F:5' -GAGGCCCCCAGGACTTTAAC-3′<br>R:5' -CTTCTTGGTGAGCGGGTTCT-3′
IL-15	Mouse	F:5' -GCTGAGTTCCACATCTAACAGC-3′<br>R:5' -AGCAAGGACCATGAAGAGGC-3′
IL-4	Mouse	F:5' -CCATATCCACGGATGCGACA-3′<br>R:5' -CGTTGCTGTGAGGACGTTTG-3′
IL-8	Mouse	F:5' -CATCCAGACCAGAGTCAGGC-3′<br>R:5' -CTTGAGTGTCACAGGGACGG-3′
IL-10	Mouse	F:5' -GCTGAAGACCCTCAGGATGC-3′<br>R:5' -GGCCTTGTAGACACCTTGGTC-3′
TGF-β	Mouse	F:5' -AGGAGACGGAATACAGGGCT-3′<br>R:5' -ATGTCATGGATGGTGCCCAG-3′
CD206	Mouse	F:5' -GGGACCTGGCAAGTATCCAC-3′<br>R:5' -CTGGGGTTCCATCACTCCAC-3′
Ym1	Mouse	F:5' -ATTTAGGAGGTGCCGTGGTC-3′<br>R:5' -AAAGCTCCTCTCAATAAGGGC-3′
Arg1	Mouse	F:5' -ACATTGGCTTGCGAGACGTA-3′<br>R:5' -ATCACCTTGCCAATCCCCAG-3′
Col4a1	Mouse	F:5' -CTCCAGGGGATCAGAGGTGA-3′<br>R:5' -GATGACCCTGGTGGTCCTTG-3′
Elastin	Mouse	F:5' -CTCACCACCCCACCTCTTTG-3′<br>R:5' -ACCCAAAGAGCACACCAACA-3′
MMP2	Mouse	F:5' -GCCCCCATGAAGCCTTGTTT-3′<br>R:5' -CTGTTGTAGGAGGTGCCCTG-3′
ZO-1	Mouse	F:5' -AGGTGAAACTCTGCTGAGCC-3′<br>R:5' -GTCACCGTCAGGAGTCATGG-3′
Occludin	Mouse	F:5' -GTCCTCCTGGCTCAGTTGAA-3′<br>R:5' -CGGACATGGCTGATGTCACT-3′
Claudin-5	Mouse	F:5' -AGTTAAGGCACGGGTAGCAC-3′<br>R:5' -GTACTTCTGTGACACCGGCA-3′
VE-cadherin	Mouse	F:5' -TGCTCACGGACAAGATCAGC-3′<br>R:5' -CTGGGGAAAGTTAGGGCCTG-3′
VEGF	Mouse	F:5' -CAGGCTGCTGATACGATGAA-3′<br>R:5' -CAGGAATCCCAGAAACAACC-3′
Vegfr1	Mouse	F:5' -GTGTCTATAGGTGCCGAGCC-3′<br>R:5' -CGGAAGAAGACCGCTTCAGT-3′
Vegfr2	Mouse	F:5' -GCTACAGAAATGGAAGGCCC-3′<br>R:5' -TGGTGAGGATGACCGTGTAG-3′

Gene Name	Species	Sequence
CD31	Mouse	F:5' -CATTGAGGCGCAGGACCACGTG-3′
		R:5' -GAAGGACTCCTGCACGGTGACG-3′
tie2	Mouse	F:5' -TCGGAGGGGGAAAGATGCTA-3′
		R:5' -ACTGCACAGCTGGTTCTTCT-3′
Ang-2	Mouse	F:5' -CTACTCGCTCAAGGCCACAA-3′
		R:5' -GTGCAGGCTTAAGTGATGCG-3′
Ki67	Mouse	F:5' -TGTGACATTCGTATCCAGCTG-3′
		R:5' -GGGACTTTCCTGGAAATTCTG-3′
β-actin	Mouse	F:5' -CCACCATGTACCCAGGCATT-3′
		R:5' -CAGCTCAGTAACAGTCCGCC-3′

Table S2. The specific RNA sequece of siRNA.

siRNA name	Sequence
IL-10-mouse-180	F:5'-GCUGGACAACAUACUGCUATT-3′
	F:5'-UAGCAGUAUGUUGUCCAGCTT-3′
IL-10-mouse-150	F:5'-GGUGAAGACUUUCUUUCAATT-3′
	F:5'-UUGAAAGAAAGUCUUCACCTT-3′
IL-10-mouse-18	F:5'-GCUAUGCUGCCUGCUCUUATT-3′
	F:5'-UAAGAGCAGGCAGCAUAGCTT-3′[i]

Note:180-siRNA, 150-siRNA, and 18-siRNA were designed to target IL-10 mRNA (RefSeq: NM_010548.2) at the 180th, 150th, and 18th nucleotide positions, respectively. Each siRNA targeted an 18-nucleotide sequence starting from the corresponding site.
